# Nanographene Oxide Attenuates Acute GVHD by Modulating Macrophage Polarization in a Xenogeneic Mouse Model

**DOI:** 10.1002/advs.202504569

**Published:** 2025-09-23

**Authors:** Aaron Yu, Hyun Sung Park, Dong‐Hoon Chae, Jae Han Park, Jiyoung Heo, Keonwoo Cho, Jiho Kim, Hyewon Lee, Sueyeon Jee, Chanwoo Kim, Soon Won Choi, Jaechul Ryu, Eun‐Hye Hur, Yunsuk Choi, Eun‐Ji Choi, Mi‐Kyung Oh, Hwa‐Yong Lee, Je‐Hwan Lee, Kyung‐Rok Yu

**Affiliations:** ^1^ Department of Agricultural Biotechnology and Research Institute of Agriculture and Life Sciences Seoul National University Seoul 08826 South Korea; ^2^ Institutes of Convergence Technology INBCT Co., Ltd. Seoul 18462 Republic of Korea; ^3^ Department of Hematology Asan Medical Center University of Ulsan College of Medicine Seoul 05505 South Korea; ^4^ Biomedical Research Center Asan Medical Center Seoul 05505 South Korea; ^5^ Division of Science Education Kangwon National University Chuncheon 24341 Republic of Korea

**Keywords:** graft versus host disease, humanized mouse, macrophage, nanographene oxide, nanomedicine

## Abstract

Nanographene oxide (NGO) exhibits immunomodulatory activity and holds promise as a therapeutic agent for graft‐versus‐host disease (GVHD). In a xenogeneic GVHD mouse model, NGO administration improves survival and attenuates pathology with reduced weight loss and leukocyte engraftment, without sustained systemic toxicity. In GVHD patient‐derived peripheral blood mononuclear cells (PBMCs), NGO treatment shifts T cell subsets toward immune homeostasis by increasing naïve T cells and decreasing effector memory T cells. Integrated transcriptomic analyses of PBMCs from GVHD patients and healthy donors reveal downregulation of pro‐inflammatory and interferon‐gamma–signal transducer and activator of transcription 1 (IFN‐γ–STAT1)–associated genes, coinciding with the suppression of M1 macrophage signatures and induction of anti‐inflammatory profiles. Mechanistically, NGO inhibits STAT1 activation and biases macrophages toward an anti‐inflammatory state, independent of reactive oxygen species scavenging and lipopolysaccharide–myeloid differentiation primary response 88 (LPS–MyD88) signaling. To improve translational feasibility, NGO‐primed macrophages (NGO‐Mac) are generated, which produce higher levels of interleukin‐10 (IL‐10), inhibit helper T cell 1 (Th1) differentiation, and promote regulatory T cell (Treg) induction in an IL‐10–dependent manner. In vivo, NGO‐Mac therapy reduces M1 macrophage infiltration, increases Treg frequencies, and attenuates GVHD pathology. These findings highlight NGO and NGO‐Mac as complementary immunotherapies, while further studies on safety, biodistribution, and feasibility are necessary for translation.

## Graphical abstract

Nanographene oxide (NGO) attenuates graft‐versus‐host disease (GVHD) severity by inhibiting STAT1‐mediated M1 macrophage polarization and suppressing T‐cell activation. NGO‐primed macrophages (NGO‐Mac) further enhance regulatory T‐cell induction via IL‐10. Both NGO and NGO‐Mac therapies mitigate inflammation and immune pathology, offering cell‐free and cell‐based immunotherapy strategies for treating acute GVHD.

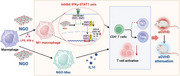

## 1 Introduction

Allogeneic hematopoietic stem cell transplantation (HSCT) is a widely utilized therapeutic approach for various malignant and nonmalignant hematological as well as immunological disorders.^[^ ^1^ ^]^ However, infusion of donor‐derived immune cells poses a significant risk, due to potential recognition of host tissues as foreign bodies. This occurs primarily through human leukocyte antigen disparities and minor histocompatibility antigen mismatches, ultimately leading to graft‐versus‐host disease (GVHD).^[^ ^2^ ^]^ Despite the advancements in GVHD prophylactic immunosuppressive therapies, acute GVHD (aGVHD) still occurs in 40–50% of transplant recipients^[^ ^3^ ^]^ and remains a leading cause of non‐relapse mortality following allogeneic HSCT.^[^ ^4^ ^]^ The standard first‐line treatment for severe acute GVHD is systemic steroid therapy, yet 35–60% of patients fail to respond to it.^[^ ^5^ ^]^ Although ruxolitinib has been approved for steroid‐refractory GVHD,^[^ ^6^ ^]^ this condition continues to be associated with high mortality, and no universally effective second‐line treatment has been established.

The pathogenesis of aGVHD involves a three‐phase cascade: initial tissue damage caused by the conditioning regimens (e.g., total body irradiation and/or chemotherapy) activates host antigen‐presenting cells (APCs); donor alloreactive T cells are subsequently stimulated and initiate the afferent phase; and finally, a pro‐inflammatory cascade damages the target organs.^[^ ^7^ ^]^ Specifically, activated host‐reactive donor T cells differentiate into helper T cell 1 (Th1) and helper T cell 17 (Th17) subsets, which result in host tissue injury via cytokine release and cytotoxic granule secretion.^[^ ^8^ ^]^ Conversely, regulatory T cells (Tregs) play an immunosuppressive role by modulating GVHD through both contact‐dependent as well as independent mechanisms.^[^ ^9^ ^]^

Besides T cells, macrophages have emerged as crucial regulators of aGVHD pathogenesis. These leukocytes play pivotal roles in both innate and adaptive immunity, owing to their remarkable heterogeneity and plasticity. Macrophages are classified into pro‐inflammatory M1 and anti‐inflammatory M2 subsets, depending on their activation state.^[^ ^10^ ^]^ M1 macrophages—typically activated by interferon‐gamma (IFN‐γ) and toll‐like receptor (TLR) ligands—engage both the IFN‐γ/signal transducer and activator of transcription 1 (STAT1) and lipopolysaccharide (LPS)/TLR4/myeloid differentiation primary response 88 (MyD88) signaling pathways and potentiate the secretion of pro‐inflammatory cytokines, such as tumor necrosis factor‐alpha (TNF‐α) and interleukin‐12 (IL‐12).^[^ ^11^ ^]^ Macrophage infiltration is a biomarker of disease progression in aGVHD,^[^ ^12^ ^]^ whereby an elevated M1/M2 ratio correlates with increased severity of grade II–IV aGVHD.^[^ ^13^ ^]^ Conversely, mesenchymal stem cell‐derived extracellular vesicles mitigate aGVHD by skewing macrophage polarization towards the M2 phenotype.^[^ ^14^ ^]^ Similarly, donor‐derived M2 macrophages have been shown to alleviate aGVHD following allogeneic HSCT in murine models.^[^ ^15^ ^]^ Although these findings suggest that macrophages represent a potential therapeutic target, the precise role of the IFN‐γ/STAT1 axis in M1 macrophage‐driven inflammation in aGVHD remains to be elucidated.

Nanographene oxide (NGO) is carbon‐based nanoparticle with excellent biocompatibility and context‐dependent immunomodulatory properties, which position it as potential therapeutic agents for inflammatory conditions, including inflammatory bowel disease and liver injury.^[^ ^16^ ^]^ NGO exhibits potent reactive oxygen species (ROS) scavenging activity due to its electron‐deficient conjugated ring structure,^[^ ^17^ ^]^ leading to reduced oxidative stress and cellular inflammation. Additionally, NGO has been shown to induce macrophage polarization toward the M2b phenotype, thereby enhancing Treg populations while suppressing Th1 and Th17 responses.^[^ ^16c^ ^]^ Recent findings suggest that NGO not only exhibits immunomodulatory properties but also possess antiviral activity, which contributes to its ability to regulate inflammatory responses in hematopoietic stem and progenitor cells.^[^ ^18^ ^]^ This potentially allows it to mitigate aberrant immune activation and hematopoietic dysregulation in patients susceptible to post‐transplantation infections.

The present study investigated the in vitro as well as in vivo therapeutic and immunosuppressive effects of NGO and NGO‐treated macrophages (NGO‐Mac) in aGVHD. Furthermore, it explored the mechanisms by which NGO and NGO‐Mac modulate macrophage polarization and subsequently attenuate aGVHD. Our findings provide novel mechanistic insights into the immunomodulatory effects of NGO on macrophages and indicate their potential as therapeutic candidates for aGVHD treatment.

## 2 Experimental Section

### 2.1 Preparation and Characterization of NGO

NGO preparations were provided by INBCT Co., Ltd (Seoul, Korea) and suspended in deionized water. NGO was synthesized from graphite via Taylor–Couette flow. The morphology of synthesized NGO particles was analyzed using Cs‐corrected high‐resolution transmission electron microscopy (HRTEM, JEM‐ARM200F, Cold FEG, JEOL Ltd, Japan) after loading onto a 400‐mesh carbon‐coated copper grid. The size distribution of NGO was determined using a CPS disc centrifuge (CPS Instruments, USA). Additionally, NGO samples prepared on sapphire wafers were examined via atomic force microscopy (AFM) in non‐contact mode (scanned area: 25 µm^2^, XE‐100, Park Systems, Korea).

### 2.2 Isolation of Human Peripheral Blood Mononuclear Cells (PBMCs)

Human peripheral blood (PB) from healthy donors was obtained from the Korean Red Cross Blood Services (Seoul, Korea) under approval from the Institutional Review Board (IRB) of Seoul National University (IRB No. E2309/002‐00). PB from patients was provided by Asan Medical Center (Seoul, Korea) under approval from the IRB No. 2023‐0700. Blood samples were collected from patients 14 days after HSCT. Acute GVHD was diagnosed and staged based on established criteria,^[^ ^19^ ^]^ and detailed clinical information for each patient used in transcriptomic analysis is provided in Table S1 (Supporting Information). Human PB mononuclear cells (hPBMCs) were isolated by density‐gradient centrifugation as described.^[^ ^20^ ^]^ CD4^+^ T cells and CD14^+^ monocytes were enriched from hPBMCs via magnetic‐activated cell sorting using human CD4^+^ or CD14^+^ MicroBead Kits (Miltenyi Biotec, Bergisch Gladbach, Germany) following the manufacturer's instructions.

### 2.3 Preparation of Macrophages

Macrophages were generated by culturing freshly isolated hCD14^+^ monocytes in RPMI1640 medium supplemented with 10% FBS and 50 ng mL^−1^ granulocyte/macrophage‐colony stimulating factor (GM‐CSF, PeproTech EC, Margravine Road, London) or macrophage‐colony stimulating factor (M‐CSF, BioLegend, San Diego, USA) for 6 days. Immature macrophages were subsequently incubated with GM‐CSF (50 ng mL^−1^), IFN‐γ (20 ng mL^−1^, BioLegend), LPS (1 µg mL^−1^, Sigma‐Aldrich, St. Louis, MO, USA) or M‐CSF (50 ng mL^−1^), IL‐4 (20 ng mL^−1^, BioLegend), and IL‐13 (20 ng mL^−1^, BioLegend) for 1–2 days to induce differentiation into M1 or M2 macrophages, respectively. To investigate the mechanism by which NGO regulates macrophage polarization, GM‐CSF–stimulated immature macrophages were treated with fludarabine (25 × 10^−6^ m; STAT1 inhibitor, MedChemExpress, Monmouth Junction, NJ, USA) or 2‐NP (45 × 10^−6^ m; STAT1 enhancer, MedChemExpress) with IFN‐γ (20 ng mL^−1^) and LPS (1 µg mL^−1^) for 2 h. To generate NGO‐macrophages (NGO‐Mac), PB‐derived CD14^+^ monocytes were cultured with M‐CSF for 6 days and subsequently treated with 20 µg mL^−1^ of NGO for 18 h.

### 2.4 Toxicity Evaluation in C57BL/6 Mice

All animal procedures were conducted following the protocol approved by the Institutional Animal Care and Use Committee of Seoul National University (SNU‐221107‐2‐5). This study is reported in accordance with ARRIVE guidelines. C57BL/6 mic (8 weeks old) were intraperitoneally (IP) injected with NGO at doses of 150, 300, or 600 µg per mouse (in saline). Body weight was monitored two to three times per week for 4 weeks. Blood samples were collected via retro‐orbital bleeding under isoflurane anesthesia at days 1, 7, 14, 21, and 28 post injection. Serum was isolated by centrifugation and analyzed for alanine aminotransferase (ALT), aspartate aminotransferase (AST), blood urea nitrogen (BUN), and creatinine using the FUJI DRI‐CHEM 3500s automated clinical chemistry analyzer (Fujifilm, Japan). For complete blood count (CBC) analysis, blood samples were collected into EDTA‐coated tubes and analyzed using an automated hematology analyzer (Sysmex XN‐V, Japan). Control animals received saline injections.

### 2.5 Xenogeneic Acute Graft‐Versus‐Host Disease Mouse Model

All animal procedures were conducted following the protocol approved by the Institutional Animal Care and Use Committee of Seoul National University (SNU‐220607‐2‐1). This study is reported in accordance with ARRIVE guidelines. NOD–scid IL2RγNULL (NSG) mice were purchased and housed in a specific pathogen‐free facility. For xGVHD induction, mice (8–13 weeks old) were irradiated with 2.4 Gy, and 24 h later, an intraperitoneal (IP) injection of 1 × 10⁶ hPBMCs in a final volume of 200 µL of sterile saline was administered. For efficacy assessment, 8‐week‐old NSG mice were IP injected with NGO at 150, 300, 600 µg in 200 µL saline on day 4 after PBMC injection, or 300 µg on day 7. Separately, ruxolitinib (MedChemExpress or Tocris Bioscience, Bristol, UK), a JAK inhibitor currently approved for steroid‐refractory GVHD was administered daily by oral gavage at 30 mg kg^−1^ day^−1^ starting from day 4 or day 7. Ruxolitinib was dissolved in DMSO and diluted in 0.5% methylcellulose (MC) solution. Treatment continued until sacrifice. Control mice received saline or vehicle (MC) only. To validate the optimal NGO treatment dose, an independent experiment was conducted using 9‐week‐old NSG mice, where only the NGO 300 µg day 4 group was compared with the PBMC‐only group under the same xGVHD induction as above. To evaluate the therapeutic efficacy of macrophage‐based cell therapy, 11–13‐week‐old NSG mice underwent xGVHD induction following the same protocol and were intravenously administered with macrophages (Mac) or NGO‐treated macrophages (NGO‐Mac) at a dose of 8 × 10^5^ cells per mouse in 200 µL saline. Control mice received saline only. GVHD severity was assessed using a scoring system incorporating four clinical parameters: weight loss, posture (hunching), mobility, and anemia, as previously described.^[^ ^21^ ^]^ Each parameter was scored from 0 (minimum) to 3 (maximum). Mice were assessed for GVHD scores thrice weekly and monitored daily. Mice reaching a GVHD score of 6/8 were sacrificed in accordance with ethical committee recommendations. Final scores for deceased animals reaching the ethical limit were retained in the dataset for subsequent time points (last value carried forward).

### 2.6 Flow Cytometry

At necropsy, PB, spleen, and bone marrow (BM) were harvested and analyzed via flow cytometry. PB was depleted of erythrocytes using RBC lysis buffer (eBioscience, San Diego, CA) following the manufacturer's instructions. Splenocytes were obtained by crushing the spleen, and BM cells were flushed from femurs and tibiae. Liver‐infiltrating cells were obtained by mincing and incubating the organs for 1 h in HBSS supplemented with 50 µg mL^−1^ DNase I (Roche, Basel, Switzerland) and 1 mg mL^−1^ collagenase A (Roche). Digestion was stopped by washing twice with PBS. Cells from all organs were washed twice with PBS containing 2% FBS. The following human‐specific antibodies were used: CD45‐APC H7, CD3‐BV510, CD4‐FITC, CD8‐BV421, CCR7‐PE, CD45RA‐APC, CD163‐BV421, CD11b‐PE, CD80‐PECy7, CD206‐APC, CD14‐APC Cy7, CD4‐APC H7, CD25‐APC, FOXP3‐PE, and IFN‐γ‐APC (all from BD Biosciences, San Jose, CA, USA). Mouse‐specific anti‐CD45‐PECy7 (BD Biosciences) was also used.

### 2.7 T‐Cell Proliferation Assay

T‐cell proliferation assays were performed as previously described.^[^ ^22^ ^]^ PBMCs were labeled with 2 × 10^−6^ m 5,6‐carboxyfluorescein succinimidyl ester (CFSE; Thermo Fisher Scientific, Waltham, MA, USA), and 2.5 × 10⁵ macrophages and 1 × 10⁵ PBMCs were added to each well with anti‐CD3/CD28 dynabeads (Gibco) and recombinant human IL‐2 (30 U mL^−1^; PeproTech). After 5 days, cells were stained with fluorescence‐labeled human monoclonal antibodies against CD45‐APC‐H7, CD3‐BV510, CD4‐APC, and CD8‐BV421 (BD Biosciences). T‐cell proliferation was analyzed via CFSE dilution using a flow cytometer (Attune Nxt, Thermo Fisher Scientific). Viable lymphocytes were gated as 7AAD‐negative cells.

### 2.8 CD4^+^ T‐Cell Differentiation

CD4⁺ T cells were stimulated with anti‐CD3/CD28 microbeads and treated with specific cytokines to induce differentiation into distinct T helper (Th) cell subsets. For Th1 differentiation, cells were cultured with IL‐2 (50 ng mL^−1^), IFN‐γ (25 ng mL^−1^), and IL‐12 (25 ng mL^−1^), while Treg differentiation was induced using TGF‐β (25 ng mL^−1^) and retinoic acid (10 × 10^−9^ m). Cytokine‐treated CD4⁺ T cells (5 × 10⁵ cells per well) were then cultured with 0, 20, or 40 µg mL^−1^ of NGO for 5 days. For intracellular cytokine staining, cells were stimulated for 5 h in RPMI medium supplemented with 10% FBS in the presence of PMA/ionomycin and GolgiPlug, a protein transport inhibitor (BD Biosciences). Th1 and Treg cells were stained with CD4‐FITC and IFN‐γ‐APC or CD4‐FITC, FOXP3‐PE, and CD25‐APC, respectively. Intracellular staining for FOXP3 and IFN‐γ was performed using the Foxp3/Transcription Factor Staining Buffer Set (eBioscience) according to the manufacturer's instructions. Flow cytometry was then used to analyze the stained cells.

### 2.9 Co‐Culture of CD4^+^ T Cells and Macrophages

For the co‐culture of CD4⁺ T cells and macrophages, a 0.4 µm Transwell system was employed (Transwell Costar, Corning, NY, USA). CD4⁺ T cells were seeded in the lower chamber with anti‐CD3/CD28 dynabeads and cultured for 5 days in the presence or absence of 50 ng mL^−1^ IL‐2, 25 ng mL^−1^ IFN‐γ, and 25 ng mL^−1^ IL‐12. Macrophages were placed in the upper chamber and treated with or without 20 µg mL^−1^ of IL‐10 neutralizing antibody. In experiments without cytokines, macrophage (Mac) or NGO‐treated macrophage (NGO‐Mac) was co‐cultured with T cells at a macrophage‐to‐T cell ratio of 1:2. After 5 days of co‐culture, T cells were stained with CD4‐FITC, FOXP3‐PE, and CD25‐APC and subsequently analyzed by flow cytometry. In experiments incorporating Th1 cytokines, Mac or NGO‐Mac was co‐cultured with T cells at a ratio of 1:4, and following 5 days, T cells were stained with either CD4‐FITC and IFN‐γ‐APC or CD4‐FITC, FOXP3‐PE, and CD25‐APC, and then analyzed by flow cytometry

### 2.10 Intracellular Reactive Oxygen Species (ROS) Measurement

To assess intracellular ROS levels, PBMC‐derived macrophages in 12‐well plates were stimulated with 50 ng mL^−1^ GM‐CSF, 20 ng mL^−1^ IFN‐γ, 1 µg mL^−1^ LPS in the presence or absence of either 20 µg mL^−1^ NGO or 10 × 10^−3^ m *N*‐acetylcysteine (NAC) for 6 h. After stimulation, the culture medium was removed and replaced with serum‐free RPMI containing 10 × 10^−6^ m DCFDA (2′,7′‐dichlorofluorescin diacetate, Sigma‐Aldrich). Cells were incubated at 37 °C for 30 min. The mean DCFDA fluorescence was analyzed immediately by flow cytometry.

### 2.11 Western Blot

Cells were lysed with RIPA buffer (Thermo Fisher Scientific) containing protease and phosphatase inhibitors (Thermo Fisher Scientific). Equal protein amounts (40 µg per sample) were subjected to SDS‐PAGE and analyzed using primary antibodies against STAT1 (#9172, Cell Signaling Technology, Danvers, MA, USA), phospho‐STAT1 (Tyr701) (58D6) (#9167, Cell Signaling Technology), MyD88 (#4283, Cell Signaling Technology), p44/42 MAPK (Erk1/2) (#4696, Cell Signaling Technology), phospho‐p44/42 MAPK (Erk1/2) (#4370, Cell Signaling Technology), p38 MAPK (#9212, Cell Signaling Technology), phospho‐p38 MAPK (#9211, Cell Signaling Technology), phospho‐IB (#9246, Cell Signaling Technology), and GAPDH (clone 0411, sc‐47724, Santa Cruz Biotechnology, Dallas, TX, USA). Bands were detected using enhanced chemiluminescence (Thermo Fisher Scientific) and imaged using the ChemiDoc XRS^+^ system (BioRad, Hercules, CA, USA). Band intensity was quantified using ImageJ software (National Institutes of Health).

### 2.12 Real‐Time PCR

Total RNA was extracted using TRIzol reagent (Invitrogen; Thermo Fisher Scientific, Inc., Carlsbad, CA, USA), and 2 µg of RNA was converted to cDNA using M‐MLV Reverse Transcriptase (Promega, Madison, WI, USA). Real‐time PCR was performed using TOPreal SYBR Green qPCR PreMIX (Enzynomics, Daejeon, Korea) and analyzed on a CFX96 Real‐Time PCR system (Bio‐Rad, Contra Costa County, CA, USA). *GAPDH* was used for normalization. Primer sequences are listed in Table S2 (Supporting Information).

### 2.13 RNA‐Sequencing

RNA sequencing (RNA‐seq) was performed by Macrogen Inc. (Seoul, South Korea) using Illumina technology as described previously.^[^ ^23^ ^]^ hPBMCs were stimulated with CD3/CD28 Dynabeads (Gibco; Thermo Fisher Scientific, Inc.) and 30 U of IL‐2 with or without 20 µg mL^−1^ of NGO for 48h. Total RNA was extracted and purified from hPBMCs using TRIzol reagent (Invitrogen).

For RNA‐seq of patient‐derived PBMCs, libraries were prepared using the SMARTer Stranded RNA‐Seq Kit (Takara Bio USA, Mountain View, CA, USA) and sequenced on an Illumina NovaSeq X platform employing a 2 × 150 bp paired‐end configuration. Libraries for normal PBMC sequencing were processed with the Illumina TruSeq Stranded mRNA Sample Preparation Kit (Illumina, San Diego, CA, USA) and sequenced on a HiSeq X Ten platform with the same 2 × 150 bp paired‐end setup. All library preparations were conducted according to the manufacturers’ protocols. After removing the adapter sequence and filtering the low‐quality reads using an in‐house script, the filtered reads were aligned to hg38 using HISAT2. The aligned reads were counted by featureCounts. For differential expression analyses, gene expression for each sample group was quantified with the edgeR R package.

For patient‐derived PBMC samples (*n* = 5 per group), differentially expressed genes (DEGs) were identified using a false discovery rate (FDR) < 0.05, except for the aGVHD versus nonGVHD comparison, for which volcano plot visualization (Figure 3B) was based on nominal *p*‐values (*p* < 0.05). For in vitro NGO‐stimulated hPBMCs, DEGs were identified based on an absolute log_2_‐fold change ≥ 1 and *p*‐value < 0.05. All heatmaps were generated using an in‐house script, and clustering analysis was performed using a hierarchical clustering method.

### 2.14 Gene Ontology (GO) Analysis and Gene Set Enrichment Analysis (GSEA)

Gene sets were identified based on gene ontology (GO) into two categories: biological processes (BP) and molecular function (MF). The significance of the gene sets was calculated using gene set enrichment analysis (GSEA v3.0, https://www.gsea‐msigdb. org/gsea/index.jsp). GO‐based trend testing was performed using Fisher's exact test.

### 2.15 Bioinformatics

Trimmed DEGs (log2‐fold change > 2.4, *p* value < 0.05) were used for pathway analysis using Qiahen's Ingenuity Pathway Analysis (IPA, Qiagen Redwood City, www.qiagen.com/ingenuity).

### 2.16 Statistical Analysis

All experiments were performed at least in triplicate. Data are presented as mean ± standard error of the mean (SEM). For comparisons between two groups, an unpaired two‐tailed student's *t*‐test was used. For comparisons involving more than two groups, one‐way ANOVA followed by Tukey's multiple comparisons test was performed. For analyses involving two independent variables, such as treatment and time, two‐way ANOVA was used followed by Tukey's multiple comparisons test. Statistical analyses were conducted using GraphPad Prism 8 (GraphPad Software Inc., San Diego, CA, USA).

## 3 Results

### 3.1 NGO Administration Does Not Induce Sustained Systemic Toxicity

To evaluate the in vivo safety of NGO, C57BL/6 mice were IP injected with 150, 300, or 600 µg of NGO and monitored for 4 weeks. Body weight monitoring revealed a transient decrease on day 1 in 300 and 600 µg groups, which subsequently returned to baseline levels. No sustained differences in weight were observed compared to the saline‐treated group (Figure S1A, Supporting Information). Serum biochemistry, including ALT, AST, BUN, and creatinine, remained within normal ranges across all groups and time points, indicating no evidence of hepatotoxicity or nephrotoxicity (Figure S1B, Supporting Information).

To further assess potential hematological toxicity, CBC analysis was performed. On day 1, platelet counts were reduced in both 300 and 600 µg groups. By week 1, the 600 µg group showed significantly decreased white blood cell (WBC) counts, mean corpuscular volume (MCV), and mean corpuscular hemoglobin concentration (MCHC) (Figure S2A, Supporting Information). A transient decline in lymphocyte counts was additionally observed at week 1 in the 600 µg group (Figure S2B, Supporting Information). However, all parameters returned to normal levels by week 2, indicating that the observed hematologic alterations were reversible. Taken together, these results suggest that while high dose of NGO (600 µg) may transiently affect hematological parameters during the early phase, no sustained systemic toxicity was observed during the 4‐week evaluation period.

### 3.2 NGO Administration Mitigates Acute GVHD Severity in a Xenogeneic Mouse Model

**Figure 1:**
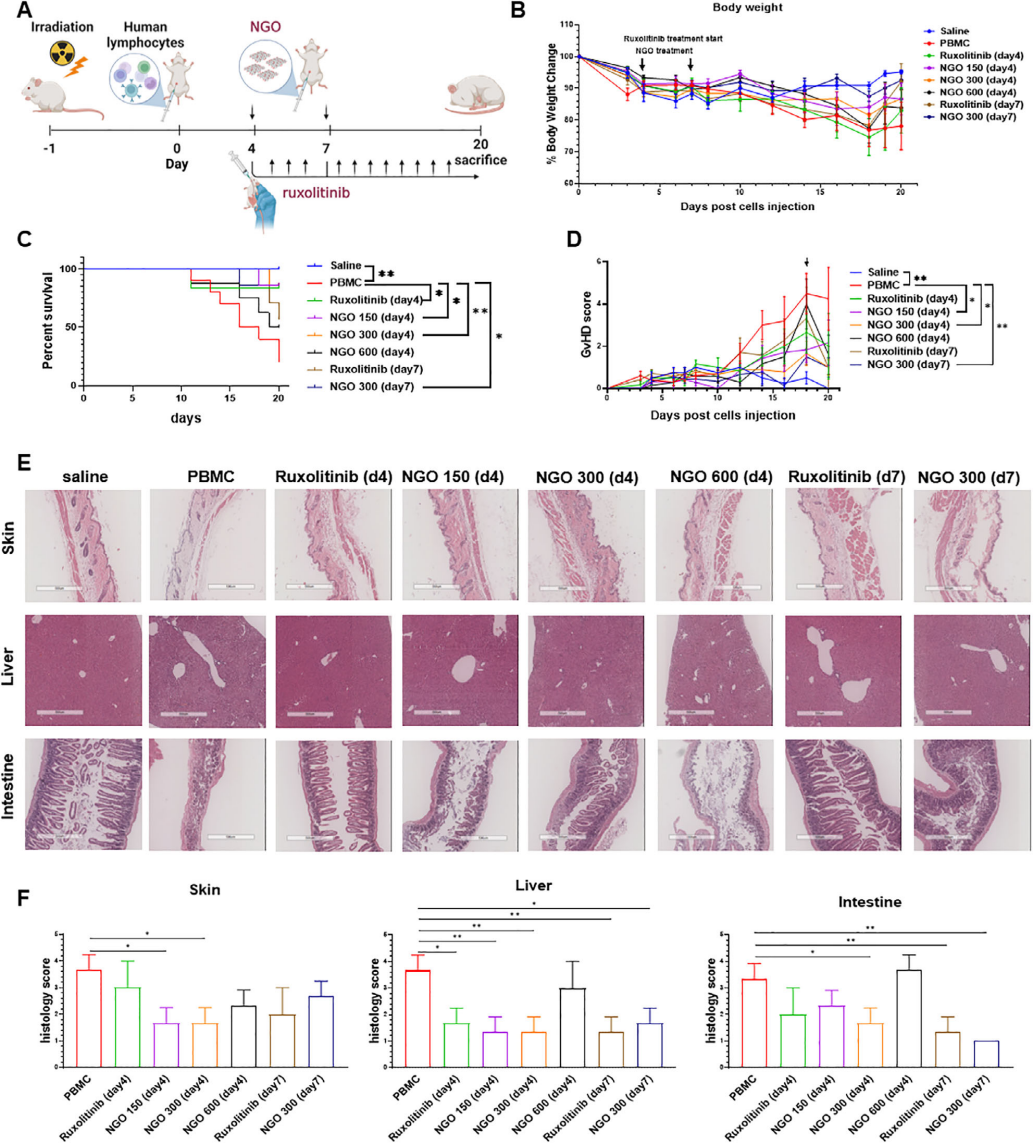
Timing‐ and dose‐dependent effects of nanographene oxide (NGO) in a xenogeneic graft‐versus‐host disease (GVHD) mouse model. Sublethally irradiated (2.4 Gy) NOD–scid IL2RγNULL (NSG) mice were injected with 1 × 10⁶ human peripheral blood mononuclear cells (PBMCs) on day 0. NGO was administered intraperitoneally (IP) at 150, 300, or 600 µg on day 4 (*n* = 7, 9, and 7, respectively) or at 300 µg on day 7 (*n* = 9). Ruxolitinib was administered by oral gavage daily from day 4 (*n* = 6) or day 7 (*n* = 7). Control groups received PBS (*n* = 4) or PBMCs only (*n* = 10). A) Experimental scheme for xenogeneic GVHD induction and treatment. Eight‐week‐old NSG mice were sublethally irradiated with 2.4 Gy one day before IP injection of 1 × 10⁶ human PBMCs on day 0. NGO (in saline) was administered IP on day 4 or day 7, and ruxolitinib was given by daily oral gavage starting from day 4 or day 7. On day 20, all surviving mice were sacrificed and peripheral blood (PB), bone marrow (BM), spleen, and organs were harvested for analysis. B) Percentage of body weight change and C) survival rate of mice in each group. D) GVHD score for each group. GVHD severity was assessed based on weight loss, fur texture, posture, and viability, with scoring conducted three times per week. Statistical comparisons between groups were performed based on GVHD scores measured on day 18, as indicated by the arrow. E,F) Representative H&E‐stained sections of skin, liver, and intestine from each group (E), and corresponding histological GVHD severity scores (F). Grades 0–2 indicate mild GVHD; grades 3–4 indicate severe GVHD. *n* = 3 mice per group. Results are presented as mean ± SEM. (^*^ *p* < 0.05, ^**^ *p* < 0.01, ^***^ *p* < 0.001).

To assess the therapeutic effects of NGO in vivo, we employed a xGVHD mouse model.^[^ ^24^ ^]^ hPBMCs were injected into sublethally irradiated immunodeficient NOD–*scid* IL2Rγ^NULL^ (NSG) mice. NGO was administered IP at doses of 150, 300, 600 µg on day 4, or at 300 µg on day 7. As a comparator, ruxolitinib was administered daily by oral gavage starting on day 4 or day 7 (**Figure** **1**A). Body weight monitoring revealed that NGO treatment at 300 µg on day 7 significantly preserved absolute body weight compared to the PBMC group, although no significant difference was observed in percent body weight change (Figure 1B, Figure S3A, Supporting Information). Mice treated with NGO (150 or 300 µg on day 4; 300 µg on day 7) or with ruxolitinib from day 4 exhibited improved survival compared to the PBMC group (Figure 1C). Consistently, GVHD clinical scores assessed on day 18 were significantly lower in the NGO150 (day 4), NGO300 (day 4), and NGO300 (day 7) groups (Figure 1D). Flow cytometric analysis of PB on day 20 revealed a significant reduction in activated T cells (effector memory T cells [TEM] + effector T cells [TE] subsets) in the NGO300 (day 4), NGO600 (day 4), and NGO300 (day 7) groups (Figure S3B, Supporting Information). Histopathological evaluation demonstrated that mice treated with NGO300 on day 4 exhibited reduced lymphocyte infiltration and tissue damage in the skin, liver, and intestine, along with significantly lower GVHD pathological scores in all three organs compared to the PBMC group (Figure 1E,F). Collectively, these results demonstrate that NGO effectively attenuates GVHD severity in vivo. Among the treatment conditions tested, a single IP administration of 300 µg NGO on day 4 post‐hPBMC injection yielded the most consistent and robust therapeutic effects, as evidenced by improvements in weight maintenance, survival, GVHD scoring, T‐cell activation, and histopathology.

To validate the reproducibility and therapeutic relevance of the optimal condition, we conducted an independent xGVHD experiment using only the NGO300 (day 4) group (Figure S4A, Supporting Information). Consistent with the earlier results, NGO‐treated mice exhibited enhanced survival (Figure S4B, Supporting Information) and improved body weight retention (Figure S4C, Supporting Information) compared to the PBMC group. Flow cytometric analysis confirmed a significant reduction in human CD45⁺ leukocyte engraftment in PB on day 27 (Figure S4D, Supporting Information). Histological analyses again showed reduced lymphocyte infiltration and tissue damage in the skin, liver, and intestine, with significantly lower GVHD pathological scores across all three organs (Figure 1F). Taken together, these results confirm that a single 300 µg dose of NGO administration on day 4 effectively mitigates GVHD severity and improves survival in the xGVHD mouse model.

### 3.3 NGO Exerts Immunomodulatory Effects on Human Immune Cell Activation

**Figure 2:**
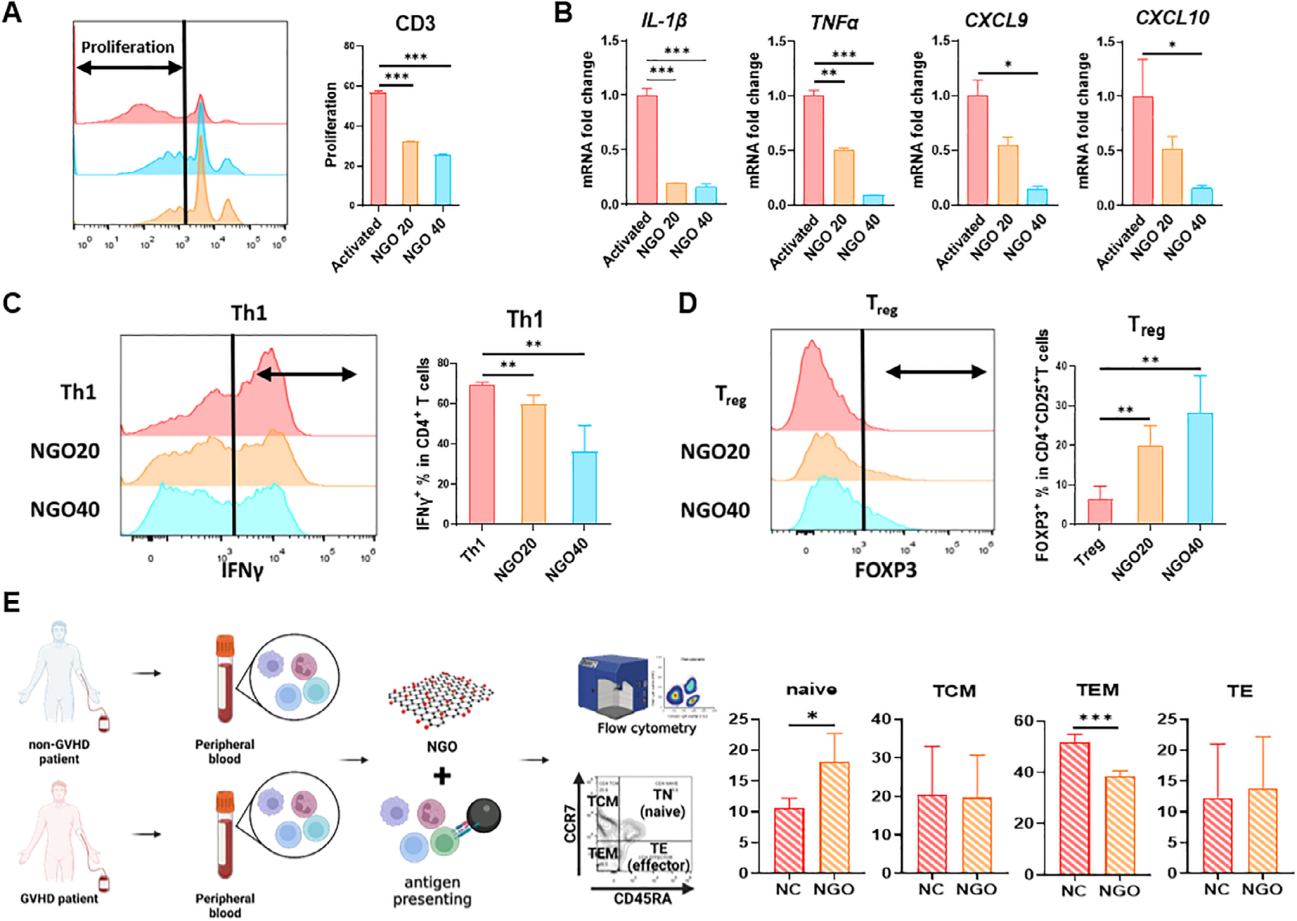
Nanographene oxide (NGO) modulates the activation of human immune cells. A) Proliferation rate of carboxyfluorescein succinimidyl ester (CFSE) labeled human peripheral blood mononuclear cells (PBMCs) after 5 days of coculture with anti‐CD3/CD28 beads, IL‐2, and varying concentrations of NGO (20, 40 µg mL^−1^). B) Relative mRNA expression levels of pro‐inflammatory factors determined by qRT‐PCR. Anti‐CD3/CD28 Dynabeads and IL‐2‐stimulated human peripheral blood mononuclear cells (hPBMCs) were co‐cultured with NGO for 48 h. C,D) CD4^+^ T cells were incubated with lineage cytokines and varying concentrations of NGO in the presence of anti‐CD3/CD28 beads and IL‐2 for 5 days. The percentages of C) Th1 (CD4^+^IFN‐γ^+^) and D) Treg (CD4^+^CD25^+^FOXP3^+^) cells were analyzed by flow cytometry and quantified. E) PBMCs from GVHD patients (*n* = 4) were incubated with anti‐CD3/CD28 Dynabeads with or without NGO for 4 days. Experimental scheme (left) and the percentage of CD4^+^ T‐cell subsets, including naïve (TN), central memory (TCM), effector memory (TEM), and effector T cells (TE) (right), are shown. Results are presented as mean ± SEM. (^*^ *p* < 0.05, ^**^ *p* < 0.01, ^***^ *p* < 0.001).

To investigate the mechanisms by which NGO mitigates GVHD, the study assessed its effects on human immune cell activation in vitro. NGO treatment led to a dose‐dependent suppression of lymphocyte proliferation (**Figure** **2**A) and significantly reduced the expression of pro‐inflammatory cytokines (*IL‐1β*, *TNFα*) and chemokines (*CXCL9*, *CXCL10*) in activated hPBMCs (Figure 2B). To further evaluate NGO's effects on T‐cell differentiation, CD4⁺ T cells were polarized into Th1 and Treg subsets using lineage‐specific cytokines. NGO treatment significantly decreased IFN‐γ‐producing Th1 (IFN‐γ⁺CD4⁺) cells (Figure 2C), whereas it enhanced the induction of FOXP3⁺CD25⁺CD4⁺ Tregs (Figure 2D). Moreover, when aGVHD patient‐derived PBMCs were cultured with NGO and CD3/CD28 dynabeads, the proportion of naïve T cells increased, whereas the effector memory T cells decreased in number (Figure 2E). These findings suggest that NGO not only suppresses T‐cell activation and proliferation, but also promotes immune regulatory mechanisms, thereby contributing to its protective effects against GVHD.

### 3.4 Transcriptome Analysis Reveals that NGO Rebalance the Immune Response in PBMCs from Patients with aGVHD

**Figure 3:**
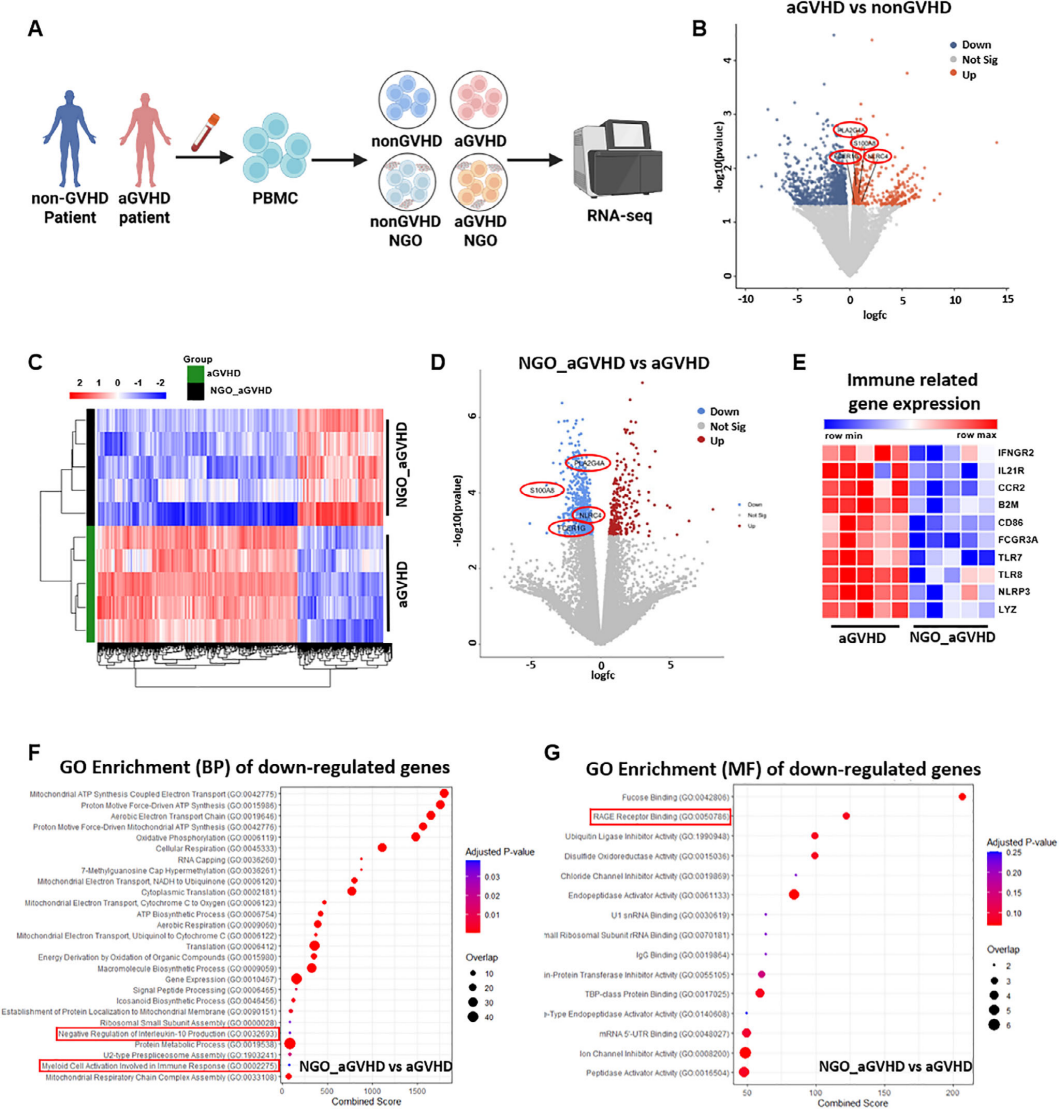
Transcriptome analysis reveals that nanographene oxide (NGO) rebalances immune response in peripheral blood mononuclear cells (PBMCs) from acute graft‐versus‐host disease (GVHD) patients. A) RNA sequencing scheme for PBMCs from acute GVHD (aGVHD) patients and allogeneic hematopoietic stem cell transplantation (HSCT) recipients without aGVHD (non‐GVHD). PBMCs were isolated from aGVHD and non‐GVHD patients, then stimulated with 20 µg mL^−1^ of NGO for 48 h in the presence of anti‐CD3/CD28 Dynabeads and IL‐2. Gene expression was analyzed (*n* = 5). B) Volcano plot showing differentially expressed genes (DEGs) between aGVHD and non‐GVHD groups. DEGs were plotted based on *p*‐values. Selected genes previously implicated in aGVHD (*S100A8*, *FCER1G*, *PLA2G4A*, and *NLRC4*) are highlighted. C) Heatmap displaying DEGs between the aGVHD NGO‐treated group and the aGVHD group. D) Volcano plot showing differentially expressed genes (DEGs) between NGO‐treated aGVHD and untreated aGVHD groups. Highlighted genes were selected based on their upregulation in aGVHD versus non‐GVHD (by P‐value) and known association with aGVHD, and were found to be downregulated upon NGO treatment. E) Heatmap illustrating immune response‐related genes in the aGVHD and aGVHD NGO‐treated groups. F,G) Gene Ontology (GO) enrichment analysis results for biological process (BP) (F) and molecular function (MF) (G) terms associated with genes downregulated in NGO‐treated aGVHD PBMCs compared to untreated aGVHD PBMCs.

To investigate the effects of NGO on immune cells in patients with aGVHD, PBMCs were obtained from individuals who either developed aGVHD or remained GVHD‐free (nonGVHD) following HSCT. PBMCs were cultured with or without NGO for 2 days, followed by RNA sequencing (**Figure** **3**A). Gene expression profiles exhibited distinct clustering based on both the aGVHD status and NGO treatment (Figure S5A, Supporting Information). No DEGs attained significant expression levels based on FDR correction in the aGVHD versus nonGVHD comparison. However, volcano plot visualization using nominal p‐values (p < 0.05) identified several immune‐related genes upregulated in aGVHD—including *S100A8*, *FCER1G*, *PLA2G4A*, and *NLRC4*. Among them, *S100A8* and *NLRC4* have previously been implicated in GVHD pathogenesis^[^ ^25^, ^26^ ^]^ whereas *FCER1G* and *PLA2G4A* are known to participate in innate immune activation and inflammatory signaling^[^ ^27^, ^28^ ^]^ (Figure 3B).

Subsequent analysis comparing NGO‐treated and untreated aGVHD PBMCs identified 779 DEGs (adjusted p < 0.05), with a significant downregulation of these immune‐related genes (Figure 3C,D). These results indicate that NGO treatment reduces pro‐inflammatory gene expression while rebalancing immune pathways in PBMCs from aGVHD patients. In addition, NGO treatment reduced the expression of multiple genes associated with immune activation and antigen presentation—such as *IFNGR2*, *CD86*, *FCGR3A*, *TLR8*, *NLRP3*, and *LYZ* (Figure 3E). GO analysis of the genes, downregulated by NGO in aGVHD PBMCs revealed enrichment in immune‐related processes, including “negative regulation of IL‐10 production” and “myeloid cell activation involved in immune response” (Figure 3F). Furthermore, “RAGE receptor binding” (Figure 3G)—a molecular function involved in amplifying inflammation through innate immune signaling—was also enriched.

To assess whether these immunomodulatory effects are consistent in nonGVHD samples, PBMCs from nonGVHD individuals treated with NGO were analyzed as well. Of the four key immune‐related genes identified above, *S100A8*, *FCER1G*, and *PLA2G4A* were also significantly downregulated in nonGVHD cases (Figure S5B,C, Supporting Information). Heatmap analysis further confirmed a reduction in the expression of additional immune‐related genes—including *CXCL11*, *JAK2*, *TLR8*, *B2M*, *STAT1*, and *LYZ*—following NGO treatment (Figure S5D, Supporting Information). Collectively, these findings demonstrate that NGO suppresses key pro‐inflammatory gene signatures in PBMCs from both aGVHD and nonGVHD individuals, thus supporting its broad immunomodulatory potential across varied donor backgrounds.

### 3.5 NGO Downregulates M1 Macrophage‐Related Genes in mRNA Transcriptome Analysis

**Figure 4:**
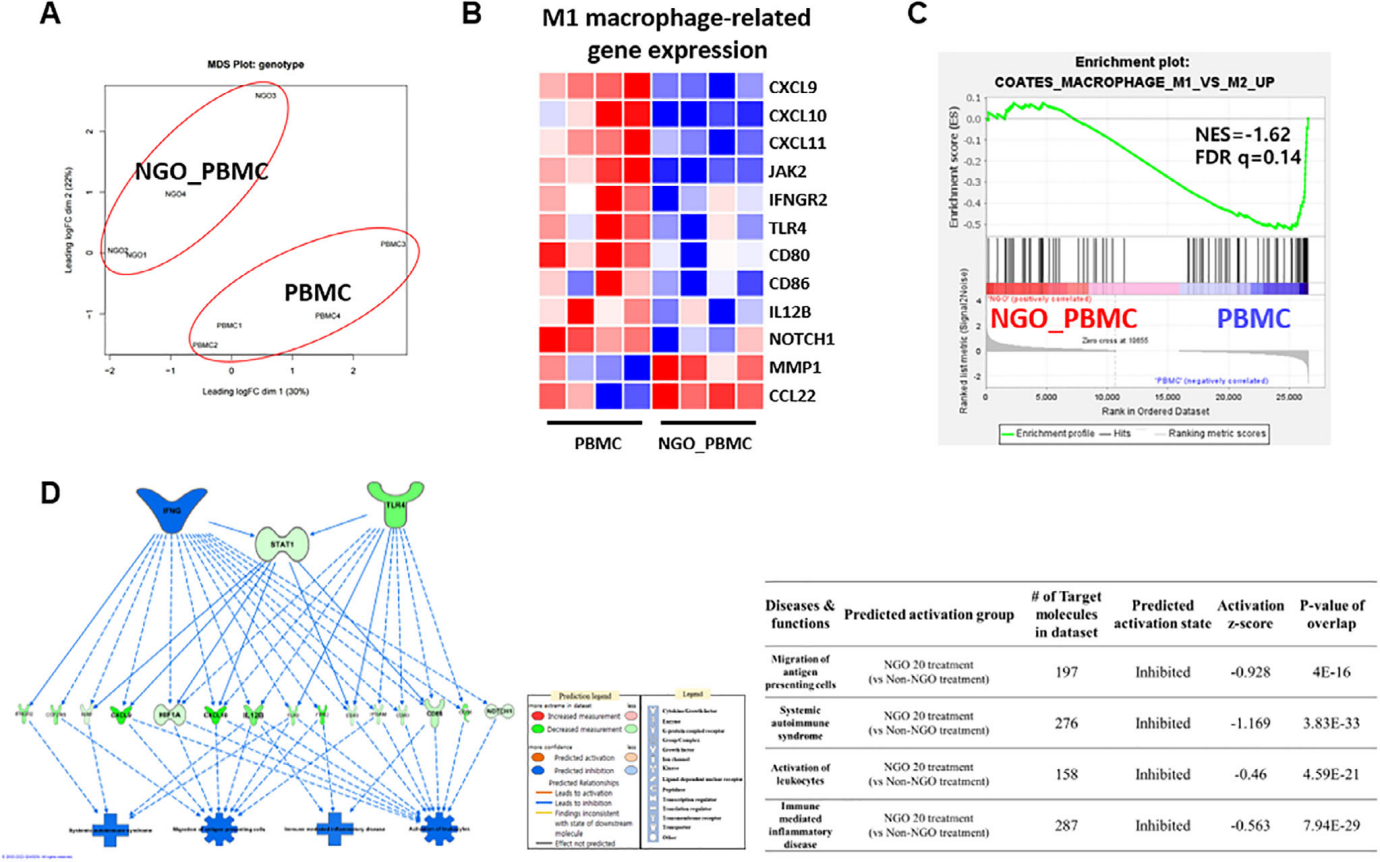
RNA expression profiles indicate that nanographene oxide (NGO) downregulates M1 macrophage‐related genes. Normal healthy peripheral blood mononuclear cells (PBMCs) were treated with 20 µg mL^−1^ of NGO for 48 h in the presence of activating anti‐CD3/CD28 Dynabeads and IL‐2, followed by gene expression analysis. A) Multidimensional scaling (MDS) plot showing the separation of PBMC and NGO‐treated samples. B) Heatmap illustrating changes in M1 macrophage‐related gene expression across all PBMC and NGO‐treated samples for differentially expressed genes. Decreased expression is indicated in blue, while increased expression is shown in red. C) Gene set enrichment analysis (GSEA) of differentially expressed genes (DEGs) enriched in upregulated genes distinguishing between M1 (pro‐inflammatory) and M2 (anti‐inflammatory) macrophage subtypes. D) Ingenuity pathway analysis (IPA) of pathways enriched in NGO‐treated PBMCs compared to untreated PBMCs.

Additional transcriptome analysis was performed using in vitro activated PBMCs from healthy donors to mitigate statistical bias from the small number of patient samples. RNA sequencing was performed on control versus NGO‐treated hPBMCs and revealed distinct clustering between the groups (**Figures** **4**A and S5E, Supporting Information). Notably, genes associated with M1 macrophage polarization and the IFN‐γ signaling pathway were downregulated by NGO exposure (Figure 4B). Moreover, GSEA identified a decrease in enrichment of the COATES_MACROPHAGE_M1_VS_M2_UP gene set following NGO exposure. This gene set comprises genes that are upregulated in M1 macrophages compared to M2 macrophages (Figure 4C).^[^ ^29^ ^]^ Additionally, ingenuity pathway analysis (IPA) predicted that the IFN‐γ and STAT1 signaling pathways that play a critical role in macrophage polarization toward the pro‐inflammatory M1 phenotype were downregulated upon treatment with NGO (Figure 4D).

### 3.6 NGO Suppresses M1 Macrophage Polarization by Selectively Inhibiting STAT1 Signaling, Independent of ROS and MyD88 Signaling

**Figure 5:**
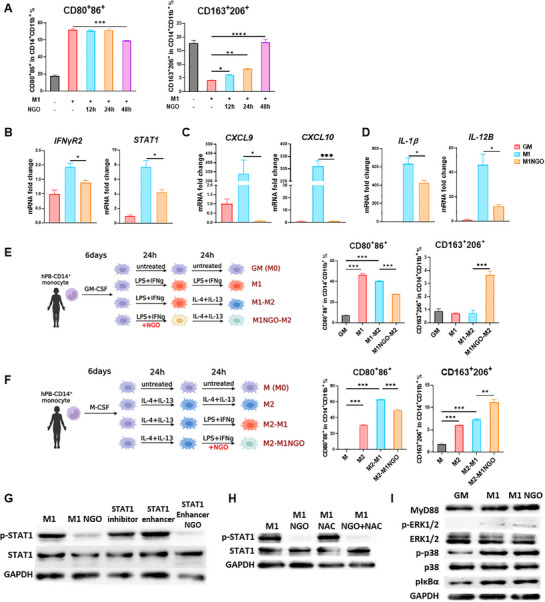
Nanographene oxide (NGO) downregulates M1 macrophage polarization via the STAT1 pathway rather than through reactive oxygen species (ROS) or LPS‐MyD88 signaling. A,B) Peripheral blood mononuclear cell (PBMC) derived macrophages were treated with NGO in the presence of LPS + IFN‐γ (M1 cytokines) for 48 h. M1 macrophage markers (CD80^+^, CD86^+^, left) and M2 macrophage markers (CD163^+^, CD206^+^, right) were analyzed using flow cytometry. B,D) Relative expression levels of IFN‐γR2 and STAT1 (B), inflammatory chemokines (C), and inflammatory cytokines (D) in M1 macrophages or NGO‐treated M1 macrophages. E,F) PBMC‐derived macrophages were repolarized from M1 to M2 (E) or M2 to M1 (F), and 20 µg mL^−1^ of NGO was administered under M1 stimulation. The expression of CD80, CD86, CD163, and CD206 in PBMC‐derived macrophages was analyzed using flow cytometry. G) Expression of STAT1 and p‐STAT1 in PBMC‐derived macrophages after 2 h of exposure to LPS + IFN‐γ in the presence of NGO, the STAT1 inhibitor (fludarabine, 25 × 10^−6^ m), or the STAT1 enhancer (2‐NP, 45 × 10^−6^ m), as assessed by Western blot. H) Expression of STAT1 and phosphorylated STAT1 (p‐STAT1) in PBMC‐derived macrophages after 30 min stimulation with LPS + IFN‐γ in the presence of NGO or 10mM NAC, as assessed by Western blot. I) Expression of MyD88, phosphorylated and total ERK1/2, p38, and phosphorylated IκBα in PBMC‐derived macrophages after 30 min stimulation with LPS + IFN‐γ, with or without NGO treatment, as assessed by western blot. Results are presented as mean ± SEM. (^*^ *p* < 0.05, ^**^ *p* < 0.01, ^***^ *p* < 0.001).

Taking into consideration the transcriptomic findings, this study further investigated NGO's role in M1 macrophage polarization. Human monocyte‐derived macrophages were differentiated into M1 macrophages using LPS and IFN‐γ, followed by NGO treatment. Flow cytometric analysis demonstrated that NGO reduced the expression of M1 surface markers CD80 and CD86, while simultaneously increasing M2 markers CD163 and CD206 in a time‐dependent manner (**Figure** **5**A). Morphologically, NGO‐treated M1 macrophages exhibited an M2‐like spindle shape, whereas untreated M1 macrophages retained a rounded appearance (Figure S6A, Supporting Information). Furthermore, transcriptomic data indicated differential expression of IFN‐γ/‐STAT1 signaling pathway genes (Figure 4B,D). RT‐PCR analysis confirmed that NGO reduced the expression of *IFN‐γR2* and *STAT1* (Figure 5B), as well as M1‐associated pro‐inflammatory chemokines (*CXCL9*, *CXCL10*) (Figure 5C) and cytokines (*IL‐1β*, *IL‐12B*) (Figure 5D) under LPS and IFN‐γ stimulation. Moreover, the M1 markers decreased and M2 polarization was promoted upon administration of M2‐inducing cytokines following M1 stimulation and NGO treatment (Figure 5E).

M1 macrophages were co‐cultured with CD4⁺ T cells to assess functional outcomes and consequently revealed that IFN‐γ production was significantly reduced with NGO treatment (Figure S6B, Supporting Information). Additionally, when M1 stimulation was followed by NGO treatment, M2 polarization was enhanced and M1 differentiation was suppressed (Figure 5F). As STAT1 phosphorylation is a key event in IFN‐γ/STAT1 signaling, we examined the effects of NGO on its regulation. Western blot analysis revealed that total STAT1 and phosphorylated STAT1 (p‐STAT1) were reduced in NGO‐treated M1 macrophages (Figure S6D, Supporting Information). Similarly, p‐STAT1 levels decreased when M2 stimulation followed M1 stimulation and NGO treatment (Figure S6E, Supporting Information). Notably, NGO more effectively inhibited STAT1 phosphorylation than the STAT1 inhibitor fludarabine, and it reversed the enhancement of STAT1 phosphorylation induced by 2‐NP (Figure 5G).

To examine whether ROS scavenging contributed to this effect, intracellular ROS levels were measured 6 h post LPS and IFN‐γ stimulation. It was observed that NGO significantly reduced ROS, although the antioxidant N‐acetylcysteine (NAC) exhibited even stronger ROS suppression (Figure S6C, Supporting Information). Nevertheless, NAC did not reduce p‐STAT1 levels at 30 min post‐stimulation, whereas NGO alone or in combination with NAC significantly reduced p‐STAT1 (Figure 5H). Therefore, these findings indicate that NGO suppresses STAT1 activation through a mechanism that is independent of ROS reduction.

This study subsequently investigated whether NGO modulates the TLR4–MyD88–NF‐κB/MAPK axis, which is another key pathway in M1 polarization. Western blot analysis of NGO‐treated macrophages revealed no changes in MyD88, ERK1/2, or p38 expression, nor in the phosphorylation of ERK1/2 or p38, or in IκBα degradation (Figure 5I). These results suggest that NGO does not interfere with canonical LPS‐MyD88 signaling. Collectively, these findings suggest that NGO selectively suppresses M0 (unpolarized) to M1 macrophage polarization by targeting the IFN‐γ/STAT1 pathway, independent of ROS scavenging or TLR4‐MyD88‐NF‐κB/MAPK signaling. This pathway specificity may underlie the relative stability of NGO‐induced reprogramming, rather than a mere transient dampening of cytokine responses.

### 3.7 NGO‐Mac Modulates T‐Cell Responses via IL‐10 Signaling

**Figure 6:**
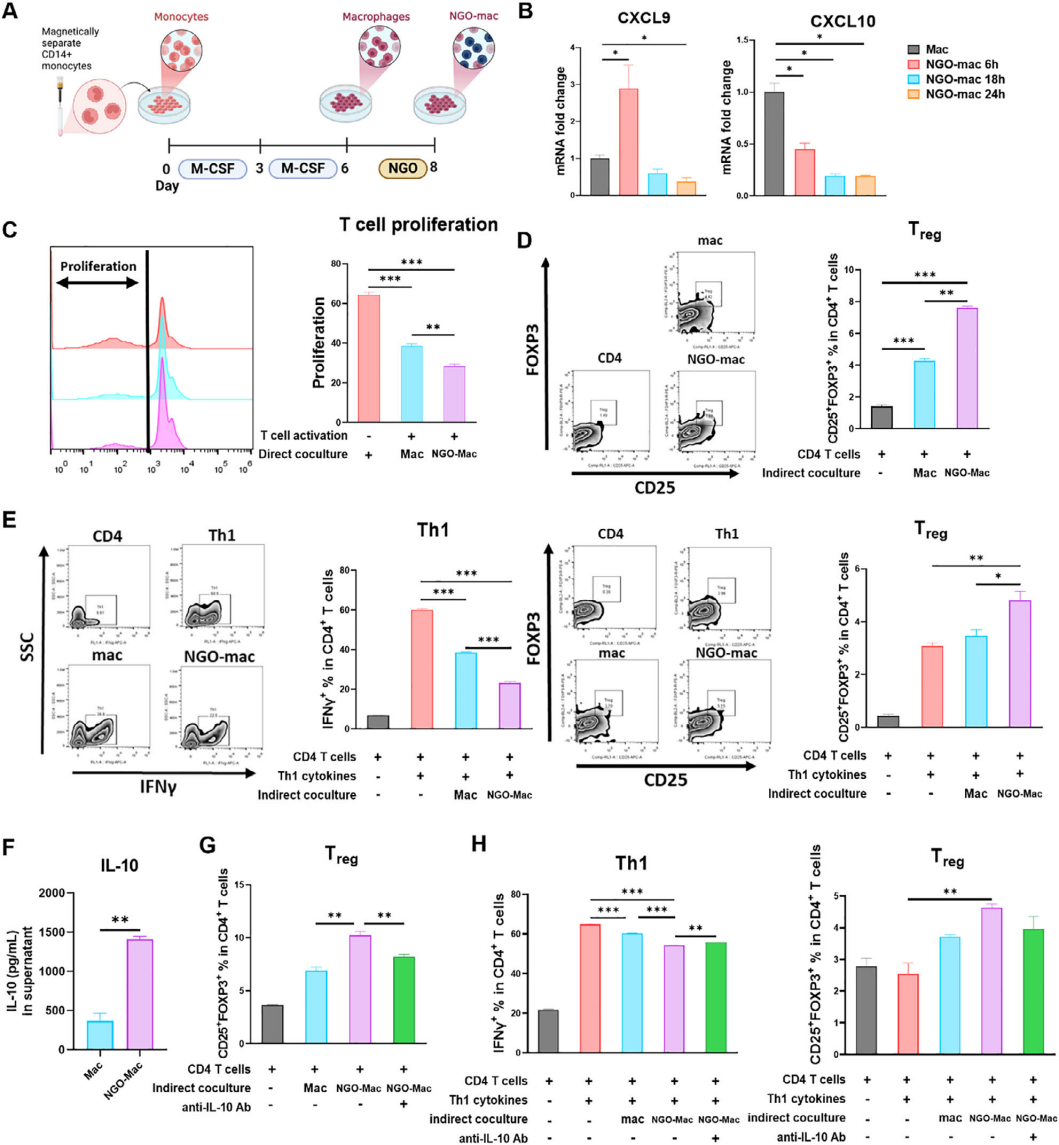
Nanographene oxide (NGO) Mac modulates T cells in vitro, with involvement of IL‐10. A) Experimental scheme for inducing NGO‐Mac. Peripheral blood mononuclear cell (PBMC) derived monocytes were treated with 50 ng mL^−1^ of macrophage colony‐stimulating factor (M‐CSF) for 6 days to generate macrophages (Mac), followed by treatment with 20 µg mL^−1^ of NGO for different durations to generate NGO‐Mac. B) Relative mRNA expression levels of inflammatory chemokines after inducing NGO‐Mac by treating macrophages with NGO for 6, 18, or 24 h. C) Proliferation rate of 5,6‐carboxyfluorescein succinimidyl ester (CFSE) labeled human PBMCs after 5 days of co‐culture with each group of cells at a 1:4 effector‐to‐target (ET) ratio in the presence of anti‐CD3/CD28 beads and IL‐2. D,E) CD4^+^ T cells were incubated without lineage‐driving cytokines at a 1:2 ET ratio (D) or with IFN‐γ and IL‐12 at a 1:4 ET ratio (E) in the presence of anti‐CD3/CD28 beads for 5 days. The percentages of CD4^+^IFN‐γ^+^ Th1 cells and CD4^+^CD25^+^FOXP3^+^ Treg cells were analyzed by flow cytometry and quantified. F) Quantification of IL‐10 in the supernatant of Mac and NGO‐Mac collected after 24–48 h of culture, as measured by ELISA. G,H) CD4^+^ T cells were incubated without lineage‐driving cytokines at a 1:2 ET ratio (G) or with IFN‐γ and IL‐12 at a 1:4 ET ratio (H) in the presence of anti‐CD3/CD28 beads for 5 days. The percentages of CD4^+^IFN‐γ^+^ Th1 cells and CD4^+^CD25^+^FOXP3^+^ Treg cells were analyzed by flow cytometry and quantified. G,H) CD4⁺ T cells were cultured under the same conditions as in (D,E), but with the addition of anti‐IL‐10 neutralizing antibody. Cells were cocultured at an E:T ratio of 1:2 without lineage‐driving cytokines (G), or at a ratio of 1:4 with IFN‐γ and IL‐12 (H). Th1 and Treg subsets were quantified by flow cytometry as described above. Results are presented as mean ± SEM. (^*^ *p*< 0.05, ^**^ *p* < 0.01, ^***^ *p* < 0.001).

Given NGO's high biocompatibility, its immunosuppressive effects require further evaluation for potential therapeutic use. To assess its immunomodulatory impact, NGO‐Mac were generated by differentiating CD14⁺ monocytes into macrophages, followed by NGO treatment (**Figure** **6**A). RT‐PCR analysis indicated that *CXCL9* and *CXCL10* levels—predominantly secreted by M1 macrophages—began decreasing at 18 h of NGO treatment (Figure 6B). Furthermore, co‐culturing CFSE‐labeled PBMCs with CD3/CD28 and IL‐2 revealed that NGO‐Mac suppressed T‐cell proliferation more effectively than NGO or untreated macrophages (Mac) (Figure 6C, Figure S6F, Supporting Information). To evaluate the effect on CD4⁺ T cells, CD4⁺ T cells and CD14⁺ monocytes were sorted from PBMCs and NGO‐Mac were generated from CD14⁺ monocytes, and subsequently co‐cultured using a transwell system. NGO‐Mac significantly increased the proportion of regulatory T cells (Tregs) compared to both the Mac group and the control group without macrophage co‐culture (Figure 6D). Moreover, under inflammatory conditions wherein helper T cells were induced toward the Th1 phenotype with IFN‐γ and IL‐12, NGO‐Mac co‐culture decreased the Th1 population while increasing Tregs (Figure 6E).

Consistent with these immunoregulatory effects, NGO‐Mac secreted markedly higher levels of IL‐10 than Mac, as determined via ELISA (Figure 6F). To ascertain whether IL‐10 mediated these effects, neutralizing antibodies against IL‐10 were added to the transwell co‐culture. IL‐10 blockade abrogated NGO‐Mac‐mediated Treg induction (Figure 6G). Furthermore, under inflammatory conditions, IL‐10 neutralization reversed the suppression of Th1 differentiation and reduced Treg expansion (Figure 6H). Overall, these results demonstrate that NGO‐Mac regulates T‐cell proliferation and differentiation via IL‐10–dependent mechanisms, thereby supporting its potential as a macrophage‐based immunoregulatory cell therapy for GVHD.

### 3.8 NGO‐Mac Attenuates aGVHD in a xGVHD Mouse Model

**Figure 7:**
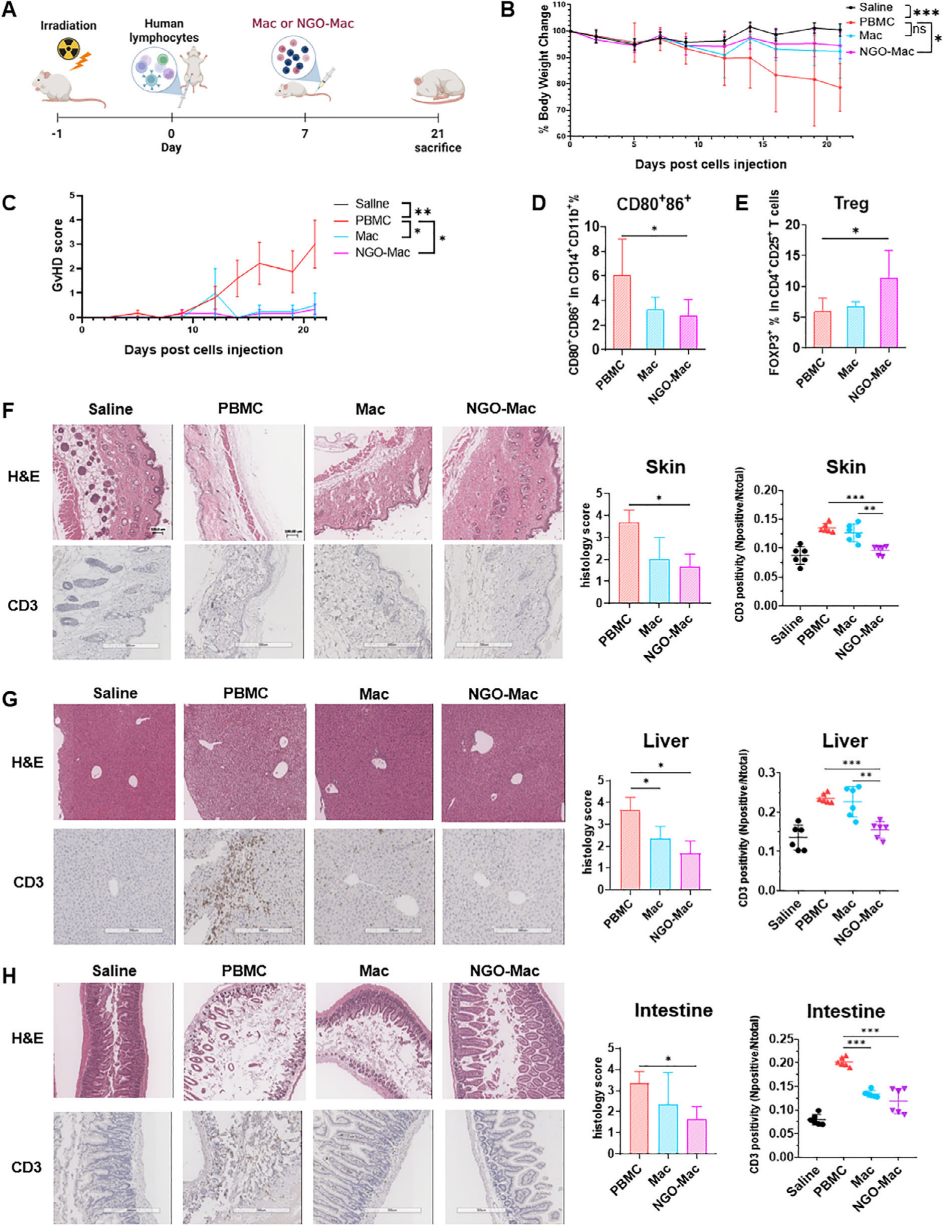
Nanographene oxide (NGO) Mac attenuates acute graft‐versus‐host disease (GVHD) in a xenogeneic GVHD mouse model. A) Experimental scheme for inducing GVHD in mice and administering Mac and NGO‐Mac. NSG mice (11–13 weeks old) were irradiated with 2.4 Gy one day prior to human peripheral blood mononuclear cells (hPBMCs) injection, and hPBMCs were intraperitoneally (IP) administered on day 0. On day 7, 8 × 10⁵ donor‐matched Mac or NGO‐Mac cells were administered intravenously. B) Percentage of body weight change monitored during the observation period. *n* = 8, 11, 5, and 6 for the saline, PBMC, Mac, and NGO‐Mac groups, respectively. C) GVHD score for each group. GVHD severity was assessed based on weight loss, fur texture, posture, and viability, with scoring conducted three times per week. D) Proportion of CD80⁺CD86⁺ human macrophages among infiltrating cells in the liver. E) Percentage of FOXP3⁺ cells within the CD4⁺CD25⁺ population in the spleen. F–H) Representative H&E staining and immunohistochemistry (IHC) staining of CD3 in skin (F), liver (G), and intestine (H) tissues from each group of mice. Histology grades 0‐2 indicate mild GVHD; grades 3‐4 indicate severe GVHD. *n* = 3 mice per group. CD3 positivity was analyzed using ImageScope software with the Positive Pixel Count v9 algorithm. Results are presented as mean ± SEM. (^*^ *p* < 0.05, ^**^ *p* < 0.01, ^***^ *p* < 0.001).

To evaluate the therapeutic potential of NGO‐Mac in vivo, Mac and NGO‐Mac were generated from CD14⁺ monocytes isolated from the same donor PBMCs used for the xGVHD model and administered to xGVHD mice (**Figure** **7**A). Mice receiving hPBMCs alone exhibited significant body weight loss, whereas those treated with NGO‐Mac maintained a stable weight (Figure 7B). Although three mice in the PBMC group and one in the Mac group died, no mortality was observed in the NGO‐Mac group (Figure S7, Supporting Information). Additionally, GVHD scores were reduced in mice treated with Mac or NGO‐Mac, compared to the PBMC‐only group (Figure 7C). Flow cytometric analysis of liver‐infiltrating immune cells further revealed a reduction in the M1 macrophage proportions in the NGO‐Mac group compared to the PBMC group (Figure 7D). Moreover, Treg frequencies were increased in the spleen of NGO‐Mac‐treated mice compared to the PBMC group (Figure 7E). Histopathological examination of skin (Figure 7F), liver (Figure 7G), and intestine (Figure 7H) tissues further supported the protective effects of NGO‐Mac. Furthermore, H&E staining and immunohistochemical (IHC) staining for CD3⁺ T cells demonstrated significantly lower GVHD pathological scores in the Mac as well as NGO‐Mac groups compared to the PBMC group, along with reduced CD3⁺ T‐cell infiltration in the NGO‐Mac group. Similarly, in the intestine, GVHD pathology scores were lower in the NGO‐Mac group, with decreased CD3⁺ T‐cell infiltration observed in the Mac, and NGO‐Mac groups.

## 4 Discussion

Previous studies on NGOs have demonstrated their potential in autoimmune disease models and inflammation regulation.^[^ ^16^, ^30^ ^]^ However, their effects on human immune cells in vivo have not been established. The present study addressed this gap by developing a xenogeneic mouse model for GVHD to evaluate the therapeutic potential of NGO in managing this disease. Our findings confirm that NGO suppresses human immune cell activation—including proliferation and differentiation—both in vitro and in vivo, thereby supporting their role in GVHD treatment. Furthermore, the sequence analysis results revealed that aGVHD is associated with distinct immune signatures. NGO treatment specifically reduced the expression of genes upregulated in aGVHD PBMCs, whereas it had no such effect in non‐GVHD PBMCs. These gene expression changes were particularly enriched in pathways related to the innate immune system, which further emphasized the immunomodulatory effects of NGO.

A key finding of this study is the ability of NGO to modulate macrophage polarization. Transcriptomic analyses revealed that NGO treatment downregulated genes associated with M1 macrophage activation, including *IFN‐γR2*, *STAT1*, and downstream pro‐inflammatory cytokines such as *CXCL9* and *IL‐1β*. This indicated the inhibition of the IFN‐γ–STAT1 signaling axis, a critical pathway in M1 macrophage‐mediated inflammation.^[^ ^11^ ^]^ To explore whether other signaling pathways might contribute to this effect, canonical innate immune signaling molecules involved in TLR–MyD88, MAPK, and NF‐κB pathways were also examined. NGO treatment did not alter the expression or phosphorylation of MyD88, pERK1/2, pp38, or pIκBα, thereby suggesting that these pathways are not involved in NGO‐mediated macrophage polarization. Furthermore, this study investigated whether NGO's inhibition of STAT1 could be attributed to its known ROS‐scavenging properties. In this regard, treatment with the ROS inhibitor NAC did not affect STAT1 phosphorylation and supported the conclusion that NGO directly inhibits the IFN‐γ–STAT1 axis, rather than acting through ROS suppression (Figure 5H). The suppression of M1 polarization was further corroborated through phenotypic and functional assessments, which demonstrated decreased expression of M1 markers (CD80, CD86) and increased expression of M2 markers (CD163, CD206) in NGO‐treated macrophages. Notably, NGO selectively reprograms M0 (unpolarized) macrophages toward an M2‐like phenotype and tends to maintain this bias even under subsequent M1‐polarizing stimuli (Figure 5A,E). This suggests that NGO may contribute to the sustenance of macrophage polarization in inflammatory settings.

The ability of NGO to modulate T‐cell responses underscores its therapeutic potential in GVHD. In vitro, NGO directly suppressed T‐cell proliferation, reduced Th1 differentiation (as indicated by decreased IFN‐γ‐producing CD4⁺ T cells), and promoted Treg expansion, which is a key mechanism for attenuating GVHD. These direct effects are consistent with previous reports demonstrating that reducing Th1 responses and enhancing Treg populations provides therapeutic benefits in GVHD.^[^ ^31^ ^]^ In addition, our findings suggest that NGO exerts indirect immunoregulatory effects by modulating macrophage polarization. NGO treatment suppressed M1 macrophage differentiation and promoted an M2‐like phenotype, which contributed to increased Treg induction. Moreover, NGO‐Mac produced elevated levels of IL‐10, which further supports this indirect pathway. Blocking IL‐10 abolished the ability of NGO‐Macs to induce Tregs and suppress Th1 responses, thus indicating that NGO‐Mac relies on an IL‐10–dependent mechanism that contributes to maintaining regulatory function even under pro‐inflammatory conditions. These findings highlight a dual mechanism by which NGO alleviates GVHD: it acts directly on T cells to suppress proinflammatory responses and also reprograms macrophages toward an anti‐inflammatory phenotype that promotes IL‐10‐dependent Treg induction. This integrated regulatory network was further validated in vivo, wherein both NGO and NGO‐Mac treatments increased Treg frequencies and reduced M1 macrophage infiltration in GVHD‐affected organs.

Despite these promising results, several questions remain to be addressed. First, while our findings provide mechanistic insight into how NGO modulates macrophage polarization, further studies are necessitated to clarify the molecular interface between NGO and immune cells. It remains unclear whether NGO interacts with specific surface receptors, is internalized through endocytic pathways, or exerts its effects through direct cytoplasmic engagement. Although our data support STAT1 as a central target of NGO activity, a more comprehensive analysis of intracellular signaling dynamics and potential secondary targets would help advance the mechanistic understanding of this mechanism and facilitate therapeutic optimization. Second, the long‐term safety and regulatory challenges associated with NGO‐based therapies must be taken into careful consideration. During this study, no significant organ toxicity or sustained hematologic abnormalities were observed over a 4‐week period following NGO administration at varying doses (150, 300, and 600 µg per head) in immunocompetent C57BL/6 mice. Mild body weight loss and a transient reduction in lymphocyte count were noted in the higher‐dose groups at the early time points, but these changes recovered to baseline levels within 1–2 weeks. These findings suggest that NGO is well tolerated at the therapeutic dose (300 µg) under physiological conditions. Consistent with our observations, prior toxicological studies demonstrated that functionalized NGO does not cause systemic toxicity in vivo, which further supports its suitability for biomedical applications even under long‐term exposure conditions.^[^ ^32^ ^]^ In addition, a recent first‐in‐human study demonstrated that controlled inhalation of graphene oxide nanosheets was safe and well tolerated in healthy volunteers, with no significant pulmonary or cardiovascular toxicity.^[^ ^33^ ^]^ Nevertheless, further studies are necessary to investigate the long‐term biodistribution, systemic clearance, and stability of NGO‐induced immune modulation. In particular, fate‐mapping studies of NGO‐primed macrophages in vivo and chronic GVHD models will be essential to evaluate their durability and translational feasibility.

Taking into consideration these safety considerations and potential challenges in obtaining regulatory approval for direct NGO administration, this study explored an alternative cell‐based therapeutic approach using NGO‐primed macrophage (NGO‐Mac). Macrophage‐based therapies have previously shown much promise in immune modulation.^[^ ^15^, ^34^ ^]^ Our findings demonstrate that NGO‐Mac exhibits an M2‐like phenotype and retains its immunosuppressive properties, which enable it to effectively suppressing Th1 differentiation and enhancing Treg expansion. The development of NGO‐Mac as a cell therapy may provide a safer and more clinically translatable approach by leveraging macrophage‐mediated delivery, while potentially reducing concerns related to direct nanoparticle exposure. Furthermore, given the heterogeneity of GVHD presentations, future studies should evaluate the efficacy of both NGO and NGO‐Mac in diverse GVHD models, including chronic GVHD and donor‐derived macrophage models.

In conclusion, our findings provide compelling evidence that NGO and NGO‐Mac effectively mitigate aGVHD by modulating macrophage polarization and T‐cell responses. A critical advantage of NGO‐based therapy is its biocompatibility and potential for clinical translation. Unlike conventional immunosuppressive therapies that broadly suppress immune function, NGO exhibits selective immunomodulation by altering macrophage polarization and promoting immune tolerance. By targeting the IFN‐γ‐STAT1 axis, NGO suppresses M1‐mediated inflammation while simultaneously enhancing Treg induction, and reducing GVHD severity without compromising essential immune functions. Moreover, the development of NGO‐Mac as a cell‐based therapy provides a promising alternative to direct NGO administration, thereby addressing safety and translational challenges while harnessing macrophages as targeted immunomodulators. Future studies exploring the precise molecular mechanisms underlying NGO‐mediated immunoregulation, as well as its long‐term safety and clinical feasibility, will be essential for advancing its therapeutic application.

## Conflict of Interest

The authors declare no conflict of interest.

## Author Contributions

A.Y., J.‐H.L., and K.‐R.Y. designed the study. A.Y., J.‐H.L., and K.‐R.Y. wrote and edited the manuscript. A.Y., H.S.P., D.‐H.C., J.H.P., J.H., K.C., J.K., H.L., S.J., C.K., S.W.C., J.R., E.‐H.H., Y.C., E.‐J.C., M.‐K.O., and H.‐Y.L. prepared the materials and conducted the experiments. A.Y., H.S.P., and J.H. conducted the statistical analysis and constructed all of the figures and tables. J.‐H.L. and K.‐R.Y. supervised the study. All the authors have read and agreed to the published version of the manuscript.

## Data Citation

1. Yu A, Yu K, Park H, Oh M, Choi S, Ryu J; 2026; Transcriptomic analysis of NGO (nano‐graphene oxide) treated human PBMCs; GEO (Gene Expression Omnibus); GSE290023; https://www.ncbi.nlm.nih.gov/geo/query/acc.cgi?acc=GSE290023

2. Yu A, Hur E, Choi S, Ryu J, Lee J, Yu K; 2026; Transcriptomic analysis of NGO (nano‐graphene oxide) treated acute GVHD or nonGVHD patients' PBMC; GEO (Gene Expression Omnibus); GSE290091; https://www.ncbi.nlm.nih.gov/geo/query/acc.cgi?acc=GSE290091

## Supporting information



Supporting Information

## Data Availability

The data that support the findings of this study are openly available in the GEO repository at https://www.ncbi.nlm.nih.gov/geo/query/acc.cgi?acc=GSE290023, reference number GSE290023 and https://www.ncbi.nlm.nih.gov/geo/query/acc.cgi?acc=GSE290091, reference number GSE290091.
