# Supplementary material for: Nanographene Oxide Attenuates Acute GVHD by Modulating Macrophage Polarization in a Xenogeneic Mouse Model
Source: Adv Sci (Weinh). 2025 Sep 23;12(45):e04569. doi: 10.1002/advs.202504569 (PMC12677671; doi:10.1002/advs.202504569)
Supplement: Supplementary file 1 — Supporting Information [file ADVS-12-e04569-s001.docx]

**Supplementary table S1.** Clinical characteristics of aGVHD and post-allo-HSCT control patients used for transcriptomic analysis

| UPN | Sex | Age | Diagnosis | Conditioning regimens | Donor | HLA match | Graft type | GVHD prophylaxis | Sample collection (week post-HSCT) | Day of GVHD onset | Affected organ* | Overall grade* |
| --- | --- | --- | --- | --- | --- | --- | --- | --- | --- | --- | --- | --- |
| No GVHD, 1 | M | 42 | AML | Bu (3d), Flu | Unrelated | 12/12 | PBSC | CSA, MTX, ATG | 2 |  | - | - |
| No GVHD, 2 | M | 63 | AML | Bu (2d), Flu | Unrelated | 12/12 | PBSC | CSA, MTX, ATG | 2 |  | - | - |
| No GVHD, 3 | M | 55 | AML | Bu (2d), Flu | Unrelated | 12/12 | PBSC | CSA, MTX, ATG | 2 |  | - | - |
| No GVHD, 4 | F | 65 | AML | Bu (2d), Flu | Unrelated | 12/12 | PBSC | CSA, MTX, ATG | 3 |  | - | - |
| No GVHD, 5 | M | 48 | AML | Bu (4d), Flu | Unrelated | 12/12 | PBSC | CSA, MTX, ATG | 2 |  | - | - |
| aGVHD, 1 | M | 61 | AMixL | Bu (2d), Flu | Unrelated | 12/12 | PBSC | CSA, MTX, ATG | 2 | 22 | GI (stage 1) | II |
| aGVHD, 2 | M | 64 | AML | Bu (2d), Flu | Unrelated | 12/12 | PBSC | CSA, MTX, ATG | 2 | 36 | Skin (stage 2) | I |
| aGVHD, 3 | F | 42 | AML | Bu (3d), Flu | Haplo-identical | 8/12 | PBSC | CSA, MTX, ATG | 2 | 70 | Skin (stage 2) | I |
| aGVHD, 4 | M | 22 | AML | Bu (3d), Flu | Haplo-identical | 8/12 | PBSC | CSA, MTX, ATG | 2 | 17 | Skin (stage 3) | II |
| aGVHD, 5 | M | 70 | AML | Bu (2d), Flu | Haplo-identical | 8/12 | PBSC | CSA, MTX, ATG | 2 | 23 | Skin (stage 3) | II |
| UPN, unique patient number; HLA, human leukocyte antigen; GVHD, graft-versus-host disease; HSCT, hematopoietic stem cell transplantation; M, male; F, female; AML, acute myeloid leukemia; AmixL, acute mixed lineage leukemia; Bu, busulfan; d, day; Flu, fludarabine; PBSC, peripheral blood stem cells; CSA, cyclosporine: MTX, methotrexate; ATG, anti-thymocyte globulin; GI, gastro-intestinal. | | | | | | | | | | | | |
| * Acute GVHD staging and grading are performed by the MAGIC criteria [19a] | | | | | | | | | | | | |

**Supplementary table S2.** Primers sequences list


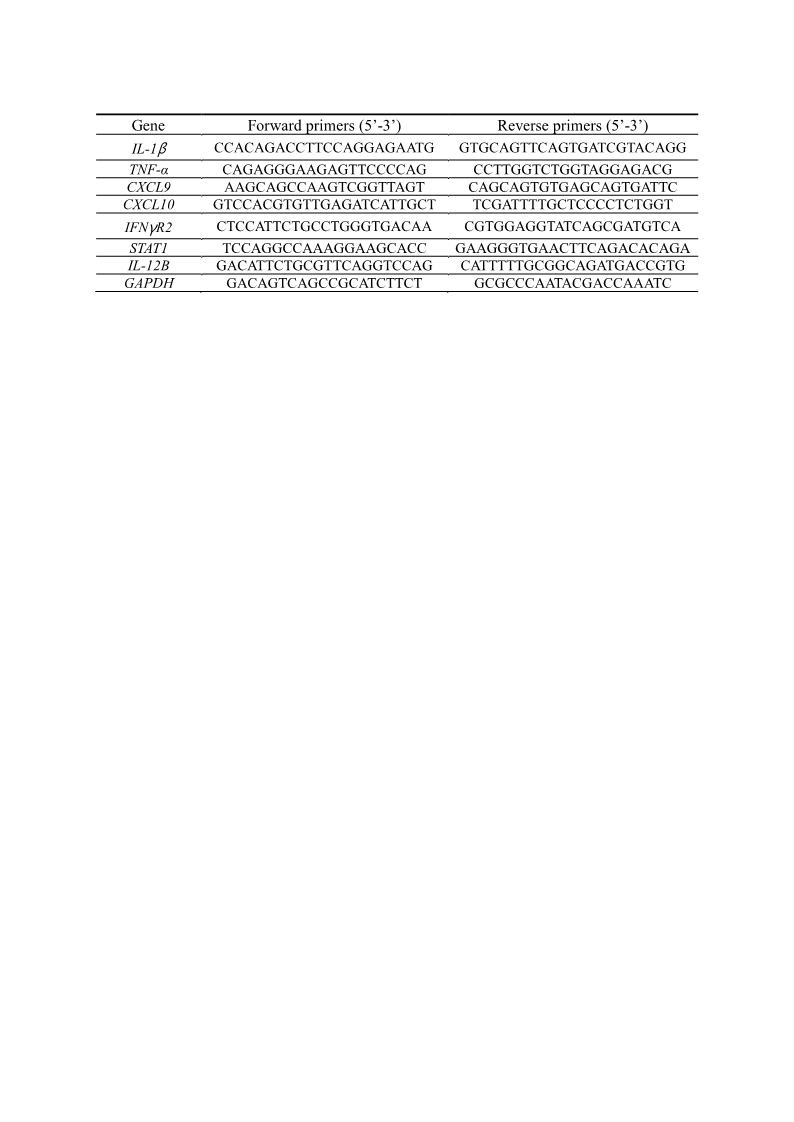


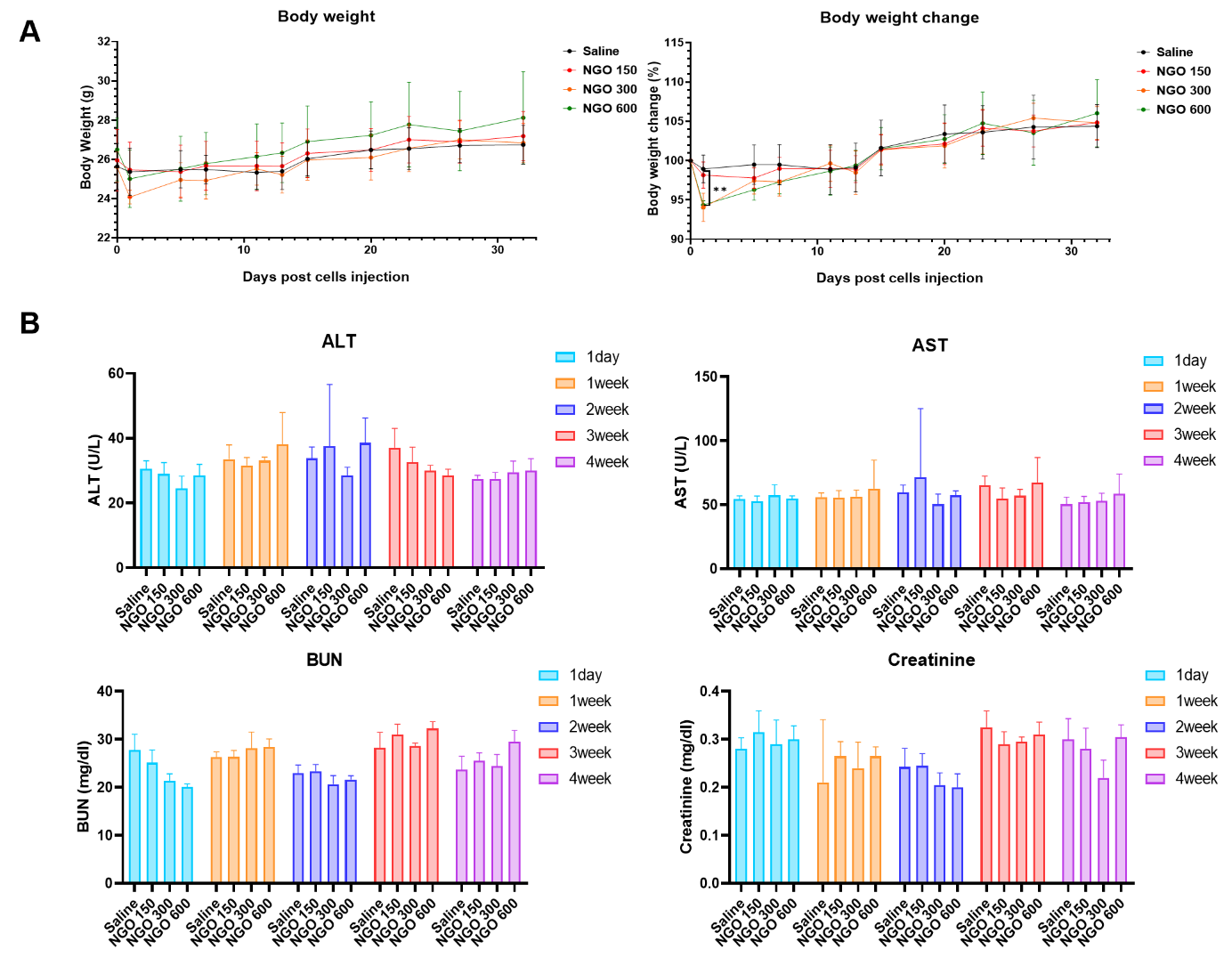


**Supplementary figure 1.** Body weight and serum biochemical parameters in C57BL/6 mice after NGO administration

(A) Body weight of C57BL/6 mice was monitored over 4 weeks following intraperitoneal administration of NGO at 150, 300, or 600 μg. Graph shows absolute body weight (left) and percent change relative to day 0 (right). (B) Serum levels of ALT, AST, BUN, and creatinine were measured at day1 and weekly up to 4 weeks after NGO injection. (*n* = 4) Results are presented as mean ± SEM. (* P < 0.05, ** P < 0.01, *** P < 0.001).

**
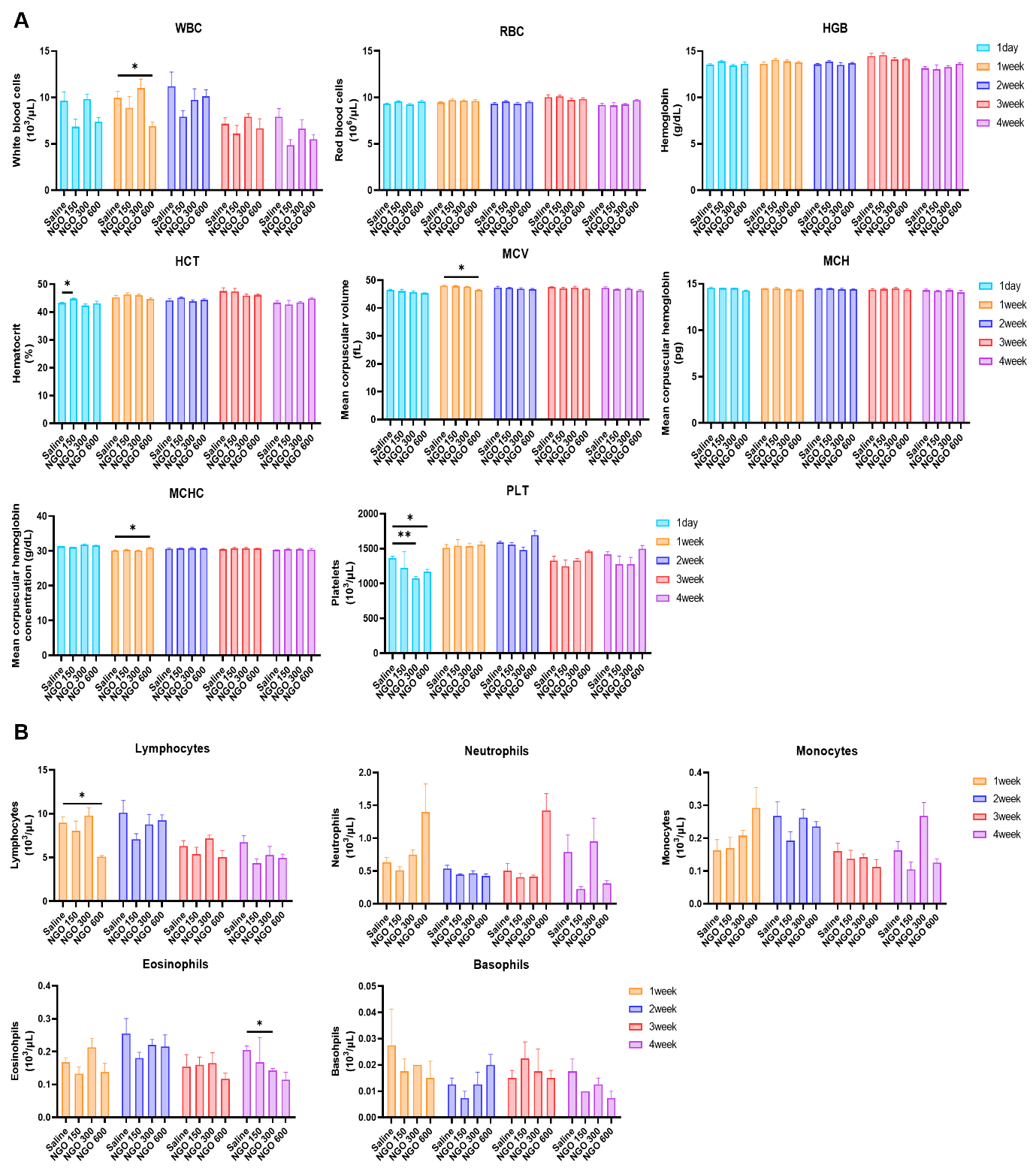
**

**Supplementary figure 2.** Hematological evaluation following NGO treatment in C57BL/6 mice

Complete blood cell counts (CBC) analysis was quantified from peripheral blood of C57BL/6 mice at day 1 and weekly up to 4 weeks after intraperitoneal injection of NGO at 150, 300, 600 μg. (A) Hematological parameters including white blood cells (WBC), red blood cells (RBC), hemoglobin (HGB), hematocrit (HCT), mean corpuscular volume (MCV), mean corpuscular hemoglobin (MCH), mean corpuscular hemoglobin concentration (MCHC), and platelets (PLT). (B) Differential leukocyte counts including lymphocytes, neutrophils, monocytes, eosinophils, and basophils. *n* = 4 mice per group; results are presented as mean ± SEM. (* P < 0.05, ** P < 0.01, *** P < 0.001).


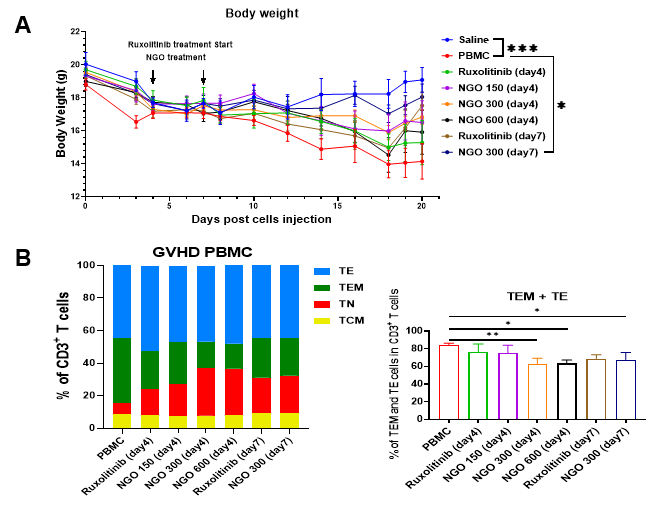


**Supplementary figure 3.** Absolute body weight and CD3^+^ T cell subset distribution in the xenogeneic GVHD mouse model following treatment with NGO or ruxolitinib

(A) Absolute body weight (g) of GVHD mice was monitored over time following PBMC injection and treatment with NGO or ruxolitinib. (B) Flow cytometry analysis of CD3^+^ T cells in peripheral blood on day 20. T cell subsets were classified into naïve (TN), central memory (TCM), effector memory (TEM), and effector T cells (TE) populations based on CCR7 and CD45RA expression. Results are presented as mean ± SEM. (* P< 0.05, ** P < 0.01, *** P < 0.001).


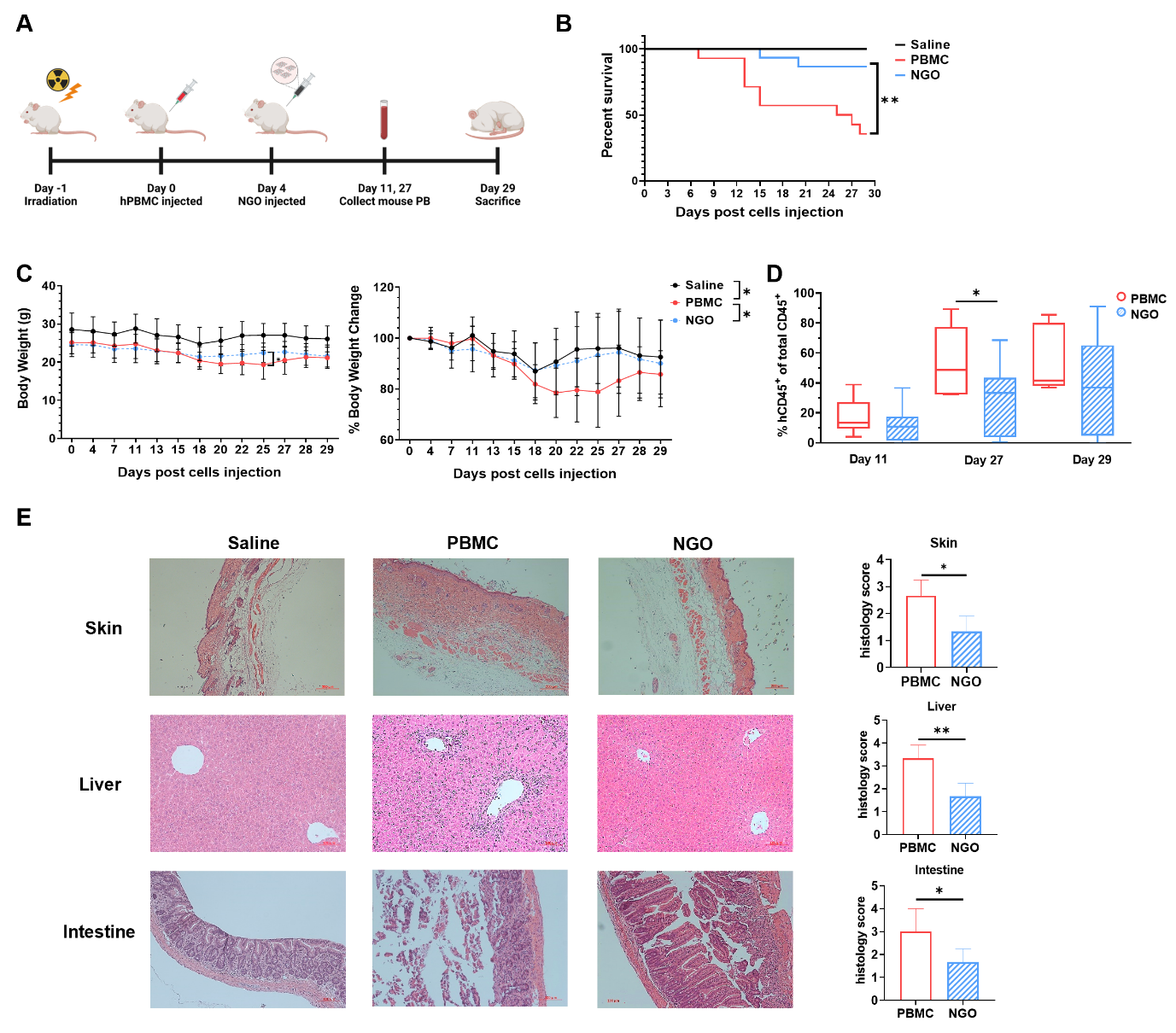


**Supplementary figure 4.** NGO Alleviates GVHD in a Xenogeneic GVHD Mouse Model

Sublethally irradiated (2.4 Gy) NSG mice received human PBMCs (hPBMCs, 1 × 10⁶) alone (PBMC, *n* = 14) or 300 μg of NGO (NGO, *n* = 15). (A) Experimental scheme for inducing GVHD in mice and administering NGO treatment. Nine-week-old NOD–scid IL2RγNULL (NSG) mice were irradiated with 2.4 Gy one day prior to hPBMC injection. hPBMCs were intraperitoneally administered on day 0, and 300 μg of NGO in PBS was administered on day 4. Peripheral blood (PB) samples were collected on days 11 and 27. On day 29, all surviving mice were sacrificed, and PB, bone marrow (BM), spleen, and tissues were harvested. (B) Percentage of body weight change and (C) survival rate of mice in each group. (D) Presence of human grafts in PB was assessed by flow cytometry for hCD45-positive cells. (hCD45% = hCD45/(hCD45+mCD45)). (E) Representative images of H&E staining for skin (50×), liver (100×), and intestine (100×) from each group, along with histological grading of GVHD severity. Grades 0–2 indicate mild GVHD; grades 3–4 indicate severe GVHD. *n* = 3 mice per group. Results are presented as mean ± SEM. (* P < 0.05, ** P < 0.01, *** P < 0.001).

**
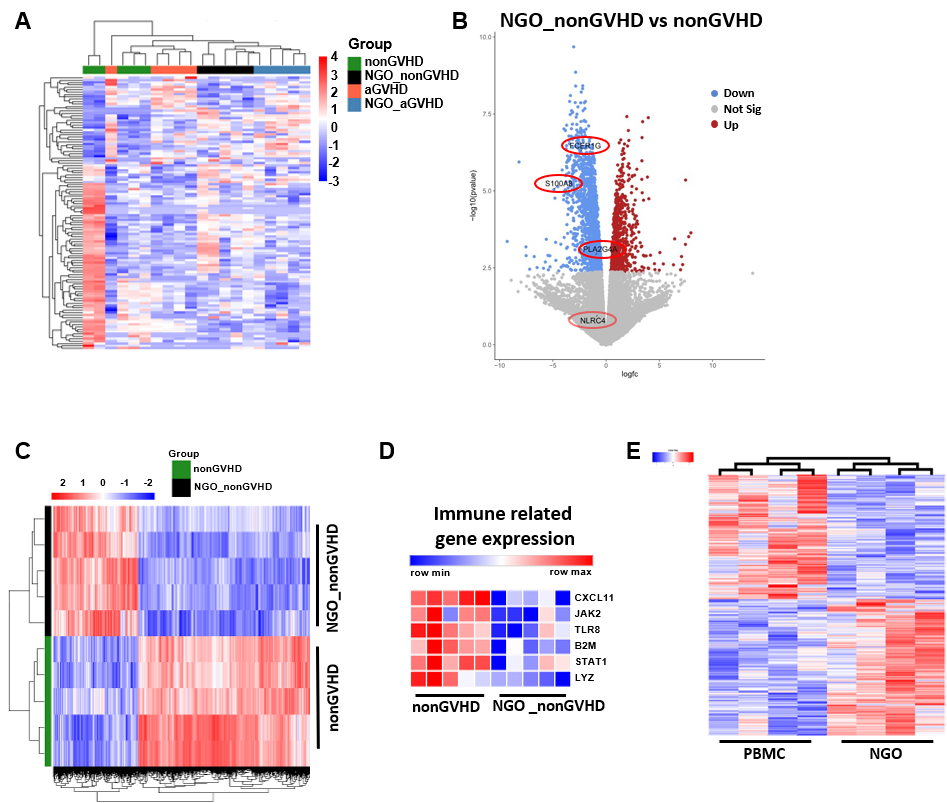
**

**Supplementary figure 5.** Transcriptomic profiles of NGO-treated PBMCs

(A) Heatmap of the top 500 differentially expressed genes (DEGs) across aGVHD, NGO_aGVHD, nonGVHD, and NGO_nonGVHD groups. DEGs were identified by one-way ANOVA with FDR correction (Benjamini–Hochberg), and gene-wise Z-score normalization was applied. (B) Volcano plot showing differentially expressed genes (DEGs) between NGO-treated nonGVHD and untreated nonGVHD groups. Highlighted genes were selected based on their upregulation in aGVHD versus non-GVHD (by P-value) and known association with aGVHD, and were found to be downregulated upon NGO treatment. (C) Heatmap corresponding to (B), further detailing the DEGs identified between nonGVHD and NGO_nonGVHD samples. (D) Heatmap displaying the expression patterns of immune response–related genes in nonGVHD and NGO_nonGVHD groups. (E) Heatmap of DEGs in PBMCs from healthy donors comparing samples with and without NGO treatment.


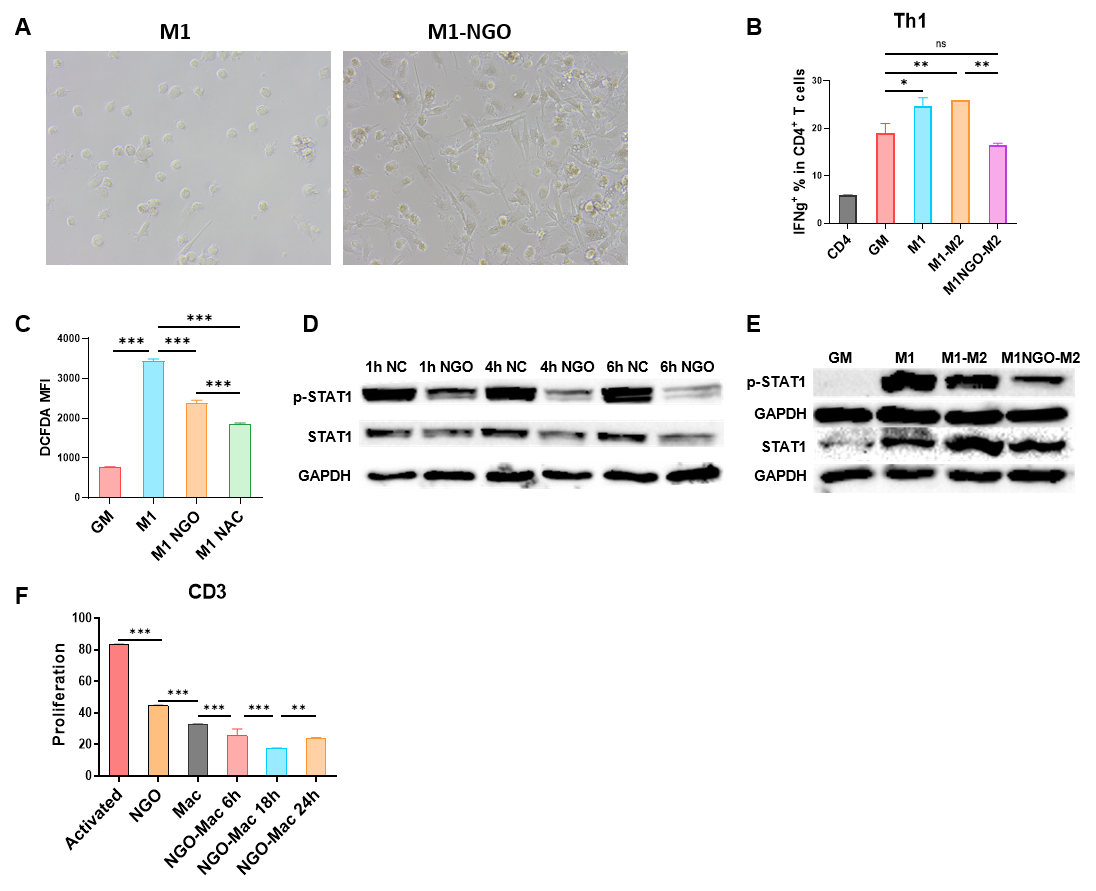


**Supplementary figure 6.** NGO exposure modulates macrophage polarization and immunoregulatory function *in* *vitro*

(A) Representative images showing the morphology of M1 macrophages after 24 hours of culture and M1 macrophages treated with NGO. (B) CD4⁺ T cells were cultured with 50 ng/mL IL-2, 25 ng/mL IFN-γ, and 25 ng/mL IL-12 in the presence of either untreated macrophages or NGO-treated macrophages at a 1:4 effector-to-target (E:T) ratio, along with anti-CD3/CD28 beads and IL-2, for five days. (C) Intracellular ROS levels in PBMC-derived macrophages treated with LPS + IFNγ for 6 h in the presence of 20 μg/mL NGO or 10 mM NAC. Cells were incubated with 10 μM DCFDA in serum-free medium for 30 min prior to flow cytometric analysis. (D) Expression of STAT1 and phosphorylated STAT1 (p-STAT1) in PBMC-derived macrophages after 1, 4, and 6 hours of exposure to LPS + IFNγ, with or without NGO treatment, as assessed by western blot. (E) Expression of STAT1 and phosphorylated STAT1 (p-STAT1) in PBMC-derived macrophages following M1 to M2 macrophage repolarization with 20 μg/mL of NGO under M1 stimulation, as assessed by western blot. (F) Proliferation of CFSE-labeled human PBMCs after five days of co-culture with untreated or NGO-treated macrophages at a 1:4 E:T ratio, in the presence of anti-CD3/CD28 beads and IL-2. Results are presented as mean ± SEM. (* P < 0.05, ** P < 0.01, *** P < 0.001).


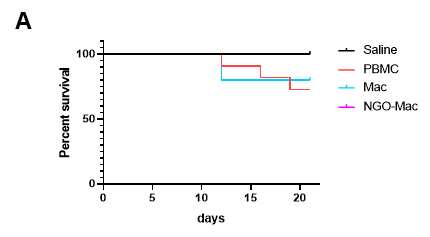


**Supplementary figure 7.** Survival rate of xGVHD mouse model following treatment with Mac and NGO-Mac

The survival rate of mice during observation period. N=8, 11, 5, and 6 for the Saline, PBMC, Mac, and NGO-Mac groups, respectively.

**Data citation**

1. Yu A, Yu K, Park H, Oh M, Choi S, Ryu J; 2026; Transcriptomic analysis of NGO (nano-graphene oxide) treated human PBMCs; GEO (Gene Expression Omnibus); GSE290023; https://www.ncbi.nlm.nih.gov/geo/query/acc.cgi?acc=GSE290023
2. Yu A, Hur E, Choi S, Ryu J, Lee J, Yu K; 2026; Transcriptomic analysis of NGO (nano-graphene oxide) treated acute GVHD or nonGVHD patients' PBMC; GEO (Gene Expression Omnibus); GSE290091; https://www.ncbi.nlm.nih.gov/geo/query/acc.cgi?acc=GSE290091
